# Versican is crucial for the initiation of cardiovascular lumen development in medaka (*Oryzias latipes*)

**DOI:** 10.1038/s41598-019-45851-3

**Published:** 2019-07-01

**Authors:** Nishant Mittal, Sung Han Yoon, Hirokazu Enomoto, Miyama Hiroshi, Atsushi Shimizu, Atsushi Kawakami, Misato Fujita, Hideto Watanabe, Keiichi Fukuda, Shinji Makino

**Affiliations:** 1 Department of Cardiology, Keio University School of Medicine, 35-Shinanomachi Shinjuku-ku, Tokyo, 160-8582 Japan; 2 Department of Interventional Cardiology, Cedars-Sinai Medical Center, 8700 Beverly Boulevard, AHSP A9229, Los Angeles, CA 90048 USA; 3 Division of Biomedical Information Analysis, Iwate Tohoku Medical Megabank Organization, Iwate Medical University, 2-1-1 Nishitokuta, Yahaba-cho, Shiwa-gun, Iwate 028-3694 Japan; 4 Department of Biological Information, Tokyo Institute of Technology, 4259 Nagatsuta, Midori-ku, Yokohama, 226-8501 Japan; 5 Department of Biological Science, Graduate School of Science, Kanagawa University, 2946 Tsuchiya, Hiratsuka-Shi, Kanagawa-Ken, 259-1293 Japan; 6 Institute for Molecular Science of Medicine, Aichi Medical University, 1-, Yazakokarimata, Nagakute, Aichi 480-1195 Japan; 7 Keio University Health Centre, 35-Shinanomachi Shinjuku-ku, Tokyo, 160-8582 Japan

**Keywords:** Disease model, Cell lineage, Medaka

## Abstract

Versican is an evolutionary conserved extracellular matrix proteoglycan, and versican expression loss in mice results in embryonic lethality owing to cardiovascular defects. However, the *in utero* development of mammals limits our understanding of the precise role of versican during cardiovascular development. Therefore, the use of evolutionarily distant species that develop *ex utero* is more suitable for studying the mechanistic basis of versican activity. We performed ENU mutagenesis screening to identify medaka mutants with defects in embryonic cardiovascular development. In this study, we described a recessive point mutation in the versican 3′UTR resulting in reduced versican protein expression. The fully penetrant homozygous mutant showed termination of cardiac development at the linear heart tube stage and exhibited absence of cardiac looping, a constricted outflow tract, and no cardiac jelly. Additionally, progenitor cells did not migrate from the secondary source towards the arterial pole of the linear heart tube, resulting in a constricted outflow tract. Furthermore, mutants lacked blood flow and vascular lumen despite continuous peristaltic heartbeats. These results enhance our understanding of the mechanistic basis of versican in cardiac development, and this mutant represents a novel genetic model to investigate the mechanisms of vascular tubulogenesis.

## Introduction

The development of the cardiovascular system is a complex and dynamic process regulated by multiple cell-cell and cell-extracellular matrix (ECM) interactions^1^, and changes in these molecular interactions can result in congenital heart diseases (CHDs) that occur in approximately 1% human live births^2^.

Embryonic ECM, that is compositionally distinct from adult ECM, contains both structural and non-structural proteins and interacts with numerous cell types. It also serves as a communication stimulus for proteins and genetic information^3–5^. Versican, a chondroitin sulfate (CS) proteoglycan that forms highly hydrated complexes with hyaluronan, is also a major component of embryonic ECM^6^. In the heart defect (*hdf*) mouse mutant, transgene insertion disrupted versican expression, resulting in embryonic lethality owing to an abnormal right ventricle, the absence of an outflow tract, and no cardiac jelly^7,8^. In another murine model, after deletion of the hyaluronan binding domain (A-subdomain of the G1-domain in versican; Vcan^Δ3/Δ3^) in a congenic C57BL/6 background, the embryos died at E10.5, similarly to the *hdf* phenotype^9^. Additionally, the targeted deletion of hyaluronan synthase 2 (*Has2*^−/−^) in a different mouse embryo resulted in a cardiac defect closely resembling that of *hdf* mice^10^. These studies indicated that hyaluronic acid and versican are essential during cardiac development; interestingly, the abovementioned mouse models also displayed unusual vascular defects, which were considered secondary effects resulting from cardiac function loss. Nevertheless, versican expression in the intima of blood vessels^11^ and upregulation in atherosclerosis lesions^12–17^ indicates that versican may play a crucial role in vasculogenesis. However, the inability to effectively monitor and manipulate the early events of murine embryonic cardiovascular development owing to *in utero* growth, limits our understanding of the underlying role of versican during the initial stages of cardiovascular development in mouse genetic mutants.

In contrast, lower vertebrates, such as zebrafish (*Danio rerio*) and medaka (*Oryzias latipes*), are ideal and versatile models to study embryonic cardiovascular development owing to their small genome size, relatively high polymorphism, easy-to-perform forward genetics, and most importantly, transparent *ex utero* development^18,19^. Their embryos, even with cardiovascular developmental defects, can survive until hatching, thereby allowing more detailed analyses. Moreover, the absence of versican orthologs in invertebrates^20^ also hints at its evolutionary relationship with the development of a closed cardiovascular system.

ENU mutagenesis (forward genetics) is an unbiased approach to identify genes essential for developmental processes and disease phenotypes. Using ENU mutagenesis, a pool of medaka mutants were generated with embryonic development defects. In the present study, we isolated a medaka mutant displaying cardiovascular defects at the embryonic stage. Since the heart development was terminated at the linear heart tube stage, we named this as linear heart tube (*lht*) mutant. These *lht* mutants displayed the absence of cardiac jelly and cardiac valves, ventricular chamber non-formation, a constricted outflow tract, and absence of a vascular lumen. Using a genetic approach, we found that these *lht* mutants showed point mutation in 3′UTR of the versican gene, resulting in a significant reduction of versican protein expression. We then demonstrated that to form an outflow tract and mature ventricular chamber, versican is vital for the migration of progenitor cells from the secondary source into the linear heart tube. Furthermore, our studies also showed the great potential of *lht* mutants as a novel model system to understand the mechanistic basis of vascular lumen formation.

## Results

### Phenotypic identification of the medaka linear heart tube (*lht*) mutant

**Figure 1:**
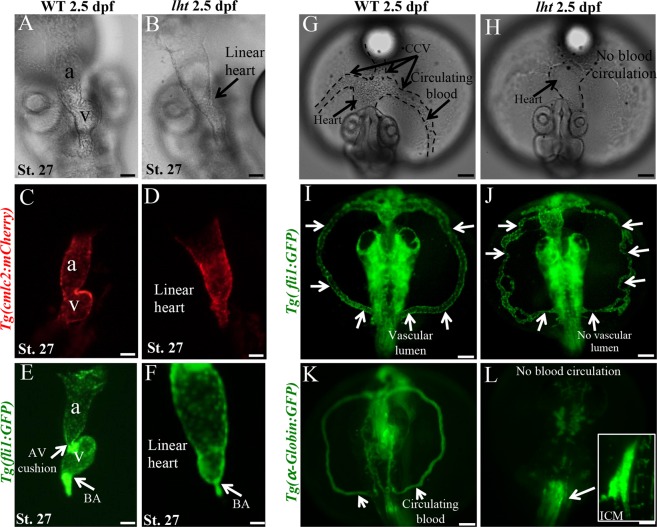
Impaired cardiac and vascular development in *lht* mutants. Phase-contrast microscopic image showing a representative cardiac phenotype in a WT and an *lht* mutant embryo at 2.5 dpf (n > 1000) (**A**,**B**). Comparison of WT and *lht* mutant cardiac development using *Tg*(*cmlc2:mCherry*) (**C**,**D**) and *Tg*(*fli1:GFP*) (**E**,**F**) transgenic medaka (n > 10). Phase-contrast microscopic image showing a representative vascular phenotype in a WT and an *lht* mutant embryo at 2.5 dpf (n > 10) (**G**,**H**). Comparison of vascular tubulogenesis (n > 10) (**I**,**J**) and blood circulation (n > 10) (**K**,**L**) in a WT and *lht* mutant embryo using *Tg*(*fli1:GFP*) and *Tg*(*α*-*globin:GFP*) medaka, respectively. Arrow in (**I**) indicates blood vessel with uniform vascular lumen. Arrow in (**J**) shows blood vessel without uniform vascular lumen. Scale bars: 50 μm (**A**–**F**), 100 μm (**G**–**L**).

The *lht* mutant was identified by screening ENU-mutagenised medaka embryos^21^. The mutation segregated as a single locus and was fully penetrant; approximately 25% (124 of 481) of the embryos obtained from a heterozygous mating developed the *lht* mutant phenotype. These recessive *lht* medaka mutants were phenotypically indistinguishable from wild-type (WT) siblings up to 40 h post-fertilization (hpf, stage 24), i.e., until the linear heart tube has developed and before the onset of blood circulation (see Supplementary Fig. S1A–D). However, between 48 and 60 hpf (stages 25–26), while the linear heart tube undergoes cardiac looping and ventricular chamber formation in WT embryos (Fig. 1AC,E; Movies 1–3), the *lht* mutants displayed the following distinguishable characteristics: enlarged sinus venosus (inflow tract) and atrium, absence of cardiac looping (Fig. 1B,D), and constricted bulbus arteriosus (BA; outflow tract) (Fig. 1F). Since cardiac looping was absent in *lht* mutant embryos, we did not observe separate atrial and ventricular chambers; therefore, we named this mutant as linear heart tube (*lht*) mutant (Movies 4–6). These *lht* mutants survived until 7 d post-fertilization (dpf), i.e., until the hatching stage, and showed continuous peristaltic beating. Therefore, we checked if there was any difference between the heart rate of *lht* mutants and WT embryos. We did not find any significant differences in the heart rate between WT and *lht* mutants, at 2 and 3 dpf (see Supplementary Fig. S1E,F).

Besides cardiac defect, *lht* mutants also displayed an absence of blood circulation. Therefore, to further characterise the *lht* mutant phenotype, we used *Tg*(*fli1:GFP*) transgenic medaka, which express GFP in endothelial cells, and found that the *lht* mutants displayed a disrupted vascular network without continuous vascular lumen, (Fig. 1I,J). Then, we used *Tg*(*α*-*globin:GFP*) transgenic medaka to determine whether *lht* mutants lack blood cell formation. We observed that the *lht* mutants lacked blood circulation (Fig. 1K,L); instead, blood cells remained intact in the inner cell mass (ICM) region (Fig. 1L, box), where primitive hematopoiesis occurs in teleosts.

Together, these results showed that *lht* mutants present severe cardiovascular defects at the embryonic stage.

### Genotypic identification of *lht* mutant medaka

**Figure 2:**
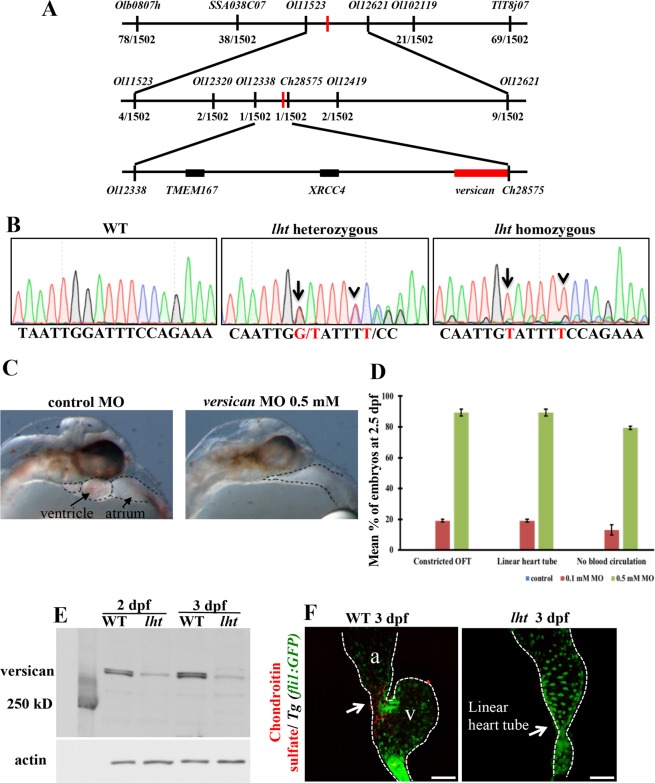
Versican: the gene responsible for the *lht* phenotype. Genetic map of *lht* (**A**). Transcript linkage map place *lht* between *Ol12338* and *Ch28575*. The numbers show the recombination events from 1,502 meiosis between the *lht* phenotype and linked genetic marker (**A**). Sequencing analysis of the genomic mutation in the versican 3′UTR. Arrow indicates the G-to-T transversion, arrowhead indicates the thymine insertion (**B**). Morpholino (MO) injection assay and quantification data (**C**,**D**). Versican MO was injected at the one-cell stage into embryos obtained from a WT cross. A morphant injected with versican MO (0.5 mM) phenocopies the *lht* mutant (**C**). Graphical representation of the dose-dependent effect of versican MO showing all the *lht* mutant phenotypes in each embryo (**D**). The results are represented as mean percentages of three independent experiments, Mean ±  SD. Western blot of versican and *actin* in WT and *lht* mutant embryos at 2 and 3 dpf (**E**). Immunohistochemistry staining for chondroitin sulfate at 3 dpf. The WT and *lht* mutant embryos were treated with chondroitinase ABC before incubation with primary antibodies (n > 10) (**F**). Arrow indicates chondroitin sulfate expression in the heart around the atrioventricular valve. Scale bar: 50 μm (**F**).

We used positional cloning to determine the genetic basis of the recessive *lht* mutant phenotype. Using genetic mapping, we found that the *lht* locus in medaka is located on chromosome 9 in a region between Ol12338 and Ch28575 (Fig. 2A). Through further fine mapping of this region, we identified a genomic sequence contig of approximately 200 kb, in which the entire region showed zero recombination with the *lht* locus (Fig. 2A). This contig contained three genes – versican, TMEM167, and XRCC4.

Since the lengths of the TMEM167 (ENSORLG00000009841) and XRCC4 (ENSORLG00000022728) genes were smaller than versican, we directly sequenced the entire TMEM167 and XRCC4 coding and untranslated regions (UTRs). However, we did not find any differences between the WT and *lht* mutant sequences of these two genes (data not shown). In contrast, the versican open reading frame encodes 3,539 amino acids and contains 15 exons spanning approximately 51 kb on chromosome 9, which was a larger size to sequence. However, since versican was already identified as a key gene in cardiac development, we first sought to understand the role of versican in medaka in detail.

The versican protein consists of three domains, namely, G1, G2 (also known as GAG domain), and G3^7^. The amino acid sequence alignment showed that G1, G2, and G3 of medaka, mouse, and human versican share 70%, 10%, and 62% homology, respectively (see Supplementary Data Set 1 and Fig. S2). Additionally, using both RT-PCR and qPCR, we found that this versican gene was expressed in multiple organs (heart, eye, gills, testis, ovary, and muscle) at the adult stage in medaka (see Supplementary Fig. S3A,B). We then examined the versican expression pattern during the various stages of embryonic development. Using whole-mount *in situ* hybridization, we found that versican was expressed prominently in the eye, heart tube, and intermediate cell mass (ICM), a region analogous to blood islands in fish^22^, until 48 hpf (see Supplementary Fig. S3C; left and middle panel); and at 72 hpf, versican was strongly expressed in the embryonic gill region (see Supplementary Fig. S3C; right panel). Next, we specifically explored the versican expression pattern at various stages of cardiac development in the medaka embryo. We found that versican mRNA was initially expressed in the entire heart tube, but by 48 hpf, versican transcripts had become localised to the outflow tract and atrioventricular cushion (see Supplementary Fig. S3D). These data, together with previously established genetic mutant mouse models^7–9^ suggested that versican is crucial for cardiac development and therefore is possibly the gene responsible for the *lht* phenotype.

To identify the mutation, we then sequenced the entire versican coding region, as well as the 5′ and 3′UTRs (untranslated regions) of the *lht* mutant, and compared the mutant and WT sequences. We found a guanine (G) to thymine (T) transversion (Fig. 2B, arrow) and a thymine insertion (Fig. 2B, arrowhead) in the 3′UTR of the versican gene. To exclude the possibility of polymorphism, we compared the versican 3′UTR sequence to those of various strains: Qurt and HNI (as the *lht* mutants were derived from these two strains), Hd-rR (because the genome-wide SNP rate between two inbred medaka strains, Hd-rR and HNI, is 3.42%, the highest SNP rate among vertebrates^23^), Kaga, and Ok-Cab. We found that the 3′UTR nucleotide sequence of the region of interest is conserved in the medaka population (see Supplementary Fig. S4A), indicating that the G–T transversion and T insertion were not owing to polymorphism. Furthermore, based on these nucleotide differences in the 3′UTR region, we genotyped 3,138 adult medaka generated from heterozygous crosses, of which 2,130 (68%) were heterozygous and 1,008 (32%) were WT. As the *lht* mutation is embryonically lethal, this 2:1 ratio was in line with the Mendelian law of inheritance, indicating that the nucleotide differences between WT and the *lht* mutant may be responsible for the *lht* phenotype.

Next, to confirm if versican was the gene responsible for the *lht* phenotype, we used a morpholino (MO) injection to knockdown versican function. Unlike the zebrafish embryo, the medaka embryo has a yolk membrane between the cell mass and yolk material and, therefore, versican MO was injected into the cytoplasm of one- or two-cell stage embryos. The morpholino for versican mRNA was designed to target the sequence in the corresponding ATG region. Three independent experiments were conducted for each concentration (0.1 and 0.5 mM). The phenotype of MO-injected embryos was evaluated at 2.5 dpf, and we found that these embryos developed dose-dependent phenotypic characteristics, such as those of the *lht* mutant (Fig. 2C; Movie 7). With an injection of 0.1 mM versican MO, 19.1% (13/68) of the embryos displayed termination of cardiac development at the linear heart tube stage and a constricted outflow tract, and 13.2% (9/68) exhibited loss of blood circulation (Fig. 2D). When we injected 0.5 mM versican MO, 89.3% (74/83) of the injected embryos again displayed the same anomalies as the *lht* mutant, and 79.4% (66/83) showed a lack of blood circulation (Fig. 2D).

To validate the efficient knockdown of versican in morphants, we used two different approaches: 1) western blot of the protein isolated from control- and versican-morphants; and 2) immunocytochemistry of medaka fibroblast cells nucleofected with versican MO.

We isolated total protein from the embryos injected with either control or versican MO. Using western blotting, we found that versican protein expression was decreased in the morphants injected with versican MO (see Supplementary Fig. S4B).

Next, we transfected the fibroblasts derived from WT medaka embryos with control MO and versican MO by nucleofection as per the protocol described in the Methods section, incubated them for 96 h and later the versican expression was compared by immunocytochemistry. From three independent experiments, the total number of cells expressing versican around the cell boundary were counted using GFP expression and nuclear staining by DAPI. In cells transfected with control MO, versican was expressed around the cell boundary (similarly to non-transfected WT cells). However, number of cells expressing versican were low when the cells were transfected with 2 mM versican morpholino (see Supplementary Fig. S4C,D). These results validated the specificity of versican to the *lht* phenotype.

The next logical step would have been to rescue the *lht* phenotype by injecting full length versican mRNA. However, since the medaka versican gene is very large (~50 kb), we were unable to clone the full length cDNA (~10 kb) to perform the rescue experiments. Therefore, we checked if injection of versican MO into another teleost, the zebrafish, could show the *lht* type of the phenotype, *i*.*e*. absence of cardiac looping, constricted outflow tract, and lack of blood circulation. Using three independent experiments with control MO and antisense MO oligonucleotides directed against zebrafish versican-a, we demonstrated that 83.5% (91/109) of the morphants showed an absence of cardiac looping; thus, depicting a linear heart tube, 78% (85/109) of the morphants showed constricted outflow tracts, and 87.1% (95/109) of the morphants lacked blood circulation (see Supplementary Fig. S4E,F; Movie 8).

Together, these results confirmed that versican is the gene responsible for the *lht* phenotype.

### Versican protein was reduced in the *lht* mutant

Once we had established that the mutation in the versican 3′UTR was responsible for the *lht* phenotype, we further analysed whether this mutation affected the mRNA or protein expression of versican.

Versican exists mainly in four isoforms (V0, V1, V2, and V3; see Supplementary Fig. S5A) through alternative splicing^7,24^. Previous studies have shown that transfecting the versican 3′UTR region alone is sufficient to alter the expression pattern of versican isoforms, thereby changing the cellular behaviour. For example, the stable transfection of a versican 3′UTR fragment in NIH3T3 fibroblast cells enhanced cellular migration^25^. Another study in transgenic mice showed that the ectopic transfection of versican 3′UTR resulted in increased expression of V0 and V1 isoforms^26^. These studies prompted us to compare the total mRNA as well as isoform specific mRNA expression of versican in WT and *lht* mutants.

Using q-PCR, we found that the total mRNA expression in the *lht* mutant was similar to that in WT (see Supplementary Fig. S5B). Then, using RT-PCR, we found that 6/7, 6/8, 7/9, and 8/9 exon boundaries were present in both WT and *lht* mutants, indicating that there was no change in the V1 and V2 isoforms (see Supplementary Fig. S5C). Moreover, we did not detect any amplicons for exon 7/8 and 6/9 boundaries, suggesting that the V0 and V3 isoforms were absent in both WT and *lht* embryos at 2.5 dpf. Together, these results indicated that the mutation in versican 3′UTR did not affect the mRNA expression of the versican gene.

Then, our next step was to compare the protein expression of versican in WT and *lht* mutants. Firstly, we performed a western blot on the total protein extracted from the whole embryos. Versican is a chondroitin sulfate proteoglycan (CSPG), which consists of a core protein and chondroitin sulfate side chains^7^. For this experiment we used a core protein (G2 domain) specific antibody. Western blot results showed variable expression patterns from two different lots of the same antibody (MC-955, Kamiya Biomedical Company; Fig. 2E and see Supplementary Fig. S4B). However, in both the cases, versican protein expression was reduced in *lht* mutants than that in WT (Fig. 2E and see Supplementary Fig. S4B).

Since the versican expression pattern was not consistent with two lots of MC-955, and none of the other commercially available versican specific antibodies (ab19345-abcam, AB1032-abcam, AB1033-abcam, V5639-100UG-Sigma, SP5182P-Acris GMBH) were suitable for medaka, we analysed the expression of chondroitin sulfate, the side chains attached to the versican core protein.

However, there are four other CSPGs besides versican: aggrecan, brevican, neurocan, and perlecan. Aggrecan is a major structural component of the cartilage and its truncation in homozygous mouse embryos causes post-natal mortality owing to respiratory failure^27^. Brevican and neurocan expression are restricted to the brain and spinal cord, and their genetic knockout mutant mouse models were viable but have shown defects in central nervous system^28–30^. Perlecan is expressed in basement membranes and cartilage, and its disruption in mouse models led to embryonic lethality owing to defective cephalic development^31–33^. All these CSPGs are not expressed in the heart during embryonic development and furthermore, the genetic knockout models of all these CSPGs did not show any cardiovascular defects. Therefore, since versican is the only CSPG expressed in the heart during cardiac development, we believe that analysing the expression pattern of a heart-specific chondroitin sulfate would reflect versican expression. It has been previously shown that CS is required for normal patterning of the atrioventricular (AV) boundary^34^. Our immunohistochemistry results showed that, in WT embryos, CS was expressed in the AV boundary of the heart. However, the entire heart tube of *lht* mutants lacked CS expression (Fig. 2F), implying that the level of versican protein was reduced in *lht* mutants.

Lastly, we analysed versican proteins at the cellular level. For this we developed an *in vitro* culture system with cells isolated from both WT and *lht* mutant medaka embryos, and performed immunocytochemistry using the G2 domain specific antibody (MC-955, Kamiya Biomedical Company). In WT cells, versican was expressed around the cell boundary, but the cells isolated from *lht* mutant embryos lacked versican expression around the periphery (see Supplementary Fig. S5D).

Together, the combined results suggested that the *lht* mutants displayed reduced versican protein expression.

### Medaka *lht* mutants present a constricted outflow tract and lack cardiac jelly

**Figure 3:**
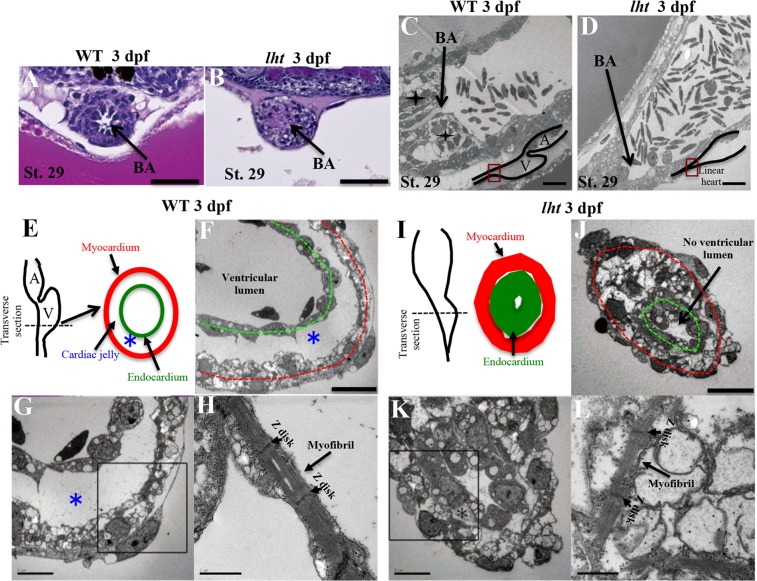
The *lht* mutant lacks cardiac jelly and displays a constricted outflow tract. Haematoxylin and eosin (H&E) staining of WT and *lht* mutant hearts at 3 dpf in the outflow tract displaying differences in cellular arrangement and lumen development (n = 3) (**A**,**B**). Scanning electron micrographs of a sagittal section of the BA, ✦ represents the cellular arrangement around the outflow tract in WT (n = 2) (**C**,**D**). Schematic representations of a transverse section of the heart from WT and *lht* mutant embryos (**E**,**I**). Black dotted line indicates parts of the sections represented in (**F**–**H** and **J**–**L**). Scanning electron micrograph of the transverse section of the ventricular chamber of a WT embryo and the distal part of the linear heart tube of an *lht* mutant embryo at 3 dpf (n = 2) (**F**,**G** and **J**,**K**). The myocardium and endocardium are represented by red and green dotted lines, respectively; *indicates cardiac jelly. Myofibril ultrastructure of the heart of WT (**H**) and *lht* mutant embryos (**L**). BA, bulbus arteriosus. Scale bars: 50 μm (**A**,**B**), 10 μm (**C**,**D**,**F**,**J**), 5 μm (**G**,**K**), and 1 μm (**H**,**L**).

Histological analysis by haematoxylin and eosin (H&E) staining, and scanning electron microscopy of the bulbus arteriosus (BA; outflow tract) showed a synchronised cellular arrangement around the BA lumen, providing elasticity to the BA in the WT case (Fig. 3A,C). In contrast, cells clustered sporadically in *lht* mutants, resulting in a constricted BA (Fig. 3B,D). To verify the BA constriction, we injected fluorescent beads into the heart of 4-dpf embryos. While the fluorescent beads spread across the vascular network in WT embryos (see Supplementary Fig. S6A,C), the beads remained within the heart tube in *lht* embryos, confirming the closure of the outflow tract (see Supplementary Fig. S6B,D).

During cardiac development, cardiac jelly is formed between the endocardial and myocardial layers of the heart tube^35^ (Fig. 3E). We confirmed the presence of thick cardiac jelly between the myocardial and endocardial cell layers in the heart tube of WT embryos (Fig. 3F,G). In contrast, the amount of cardiac jelly in the heart of the *lht* mutant was negligible (Fig. 3I,J,K). A similar arrangement of myofibrils was evident in both WT (Fig. 3H) and *lht* mutants (Fig. 3L).

Interestingly, these phenotypic characteristics of our medaka *lht* mutant were remarkably similar to *Has2*-deficient^10^ and versican-deficient heart defect^7–9^ mouse embryos, and therefore the *lht* medaka mutant represents a promising alternative vertebrate model to further investigate the role of versican during cardiac development.

### Loss of versican function impairs recruitment of progenitor cells from the secondary source to arterial pole of the heart tube

During heart development, the cardiac crescent fuses at the midline resulting in the first heart field-derived linear heart tube, which subsequently begins to beat, undergoes rightward looping and, differentiates into atrial and ventricular-specific cardiomyocytes. As development proceeds, the linear heart tube expands and recruits progenitor cells from a secondary source that comprises secondary heart field-derived cells (SHF) and cardiac neural crest cells^36,37^. Using cardiac (*cmlc2*), atrial (*amhc*), and ventricular (*vmhc*) cardiac-specific markers, we found that the heart tube of *lht* mutants was composed of both atrial- and ventricular-specific cardiomyocytes (see Supplementary Fig. S7A–D), suggesting that cardiac chamber-specific differentiation of cardiomyocytes residing in the heart tube is not altered in *lht* mutants.

**Figure 4:**
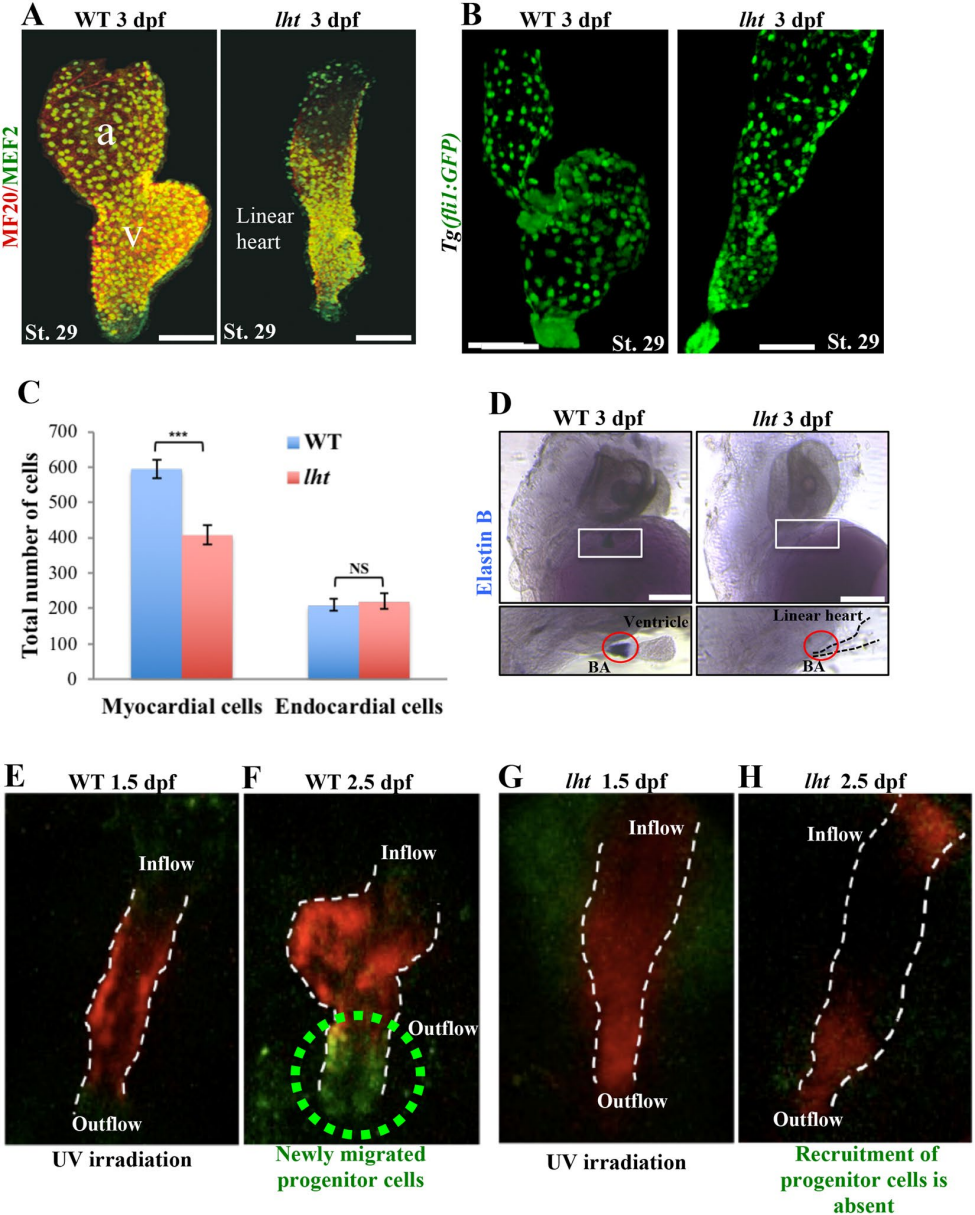
Loss of versican disrupts the migration of cardiac progenitors from the secondary source towards the linear heart tube. Cardiomyocyte cell count assay (**A**). The 3-dpf WT and *lht* mutant embryos were stained with MF20 (red) and MEF2 (green). Cells were counted using ImageJ software (n = 4). Endocardial cell count using *Tg*(*fli1:GFP*) medaka (n = 4) (**B**). Graphical representation of the total number of myocardial and endocardial cells in WT and *lht* mutant embryos (**C**). *In situ* hybridization for *elnb* expression (**D**). The WT embryo showed prominent expression of *elnb* in the outflow tract (red circle). However, *elnb* expression was absent in the outflow tract of the *lht* mutant embryo (n > 10). Photoconvertible mRNA injection assay (**E**–**H**). Kaede mRNA was injected into one-cell-stage embryos obtained from an *lht* heterozygous cross. The linear heart tube of the embryos at 1.5 dpf was irradiated with UV light, leading the differentiated cardiomyocytes to develop red fluorescence (**E**,**G**). These embryos were allowed to develop until 2.5 dpf. Furthermore, embryos displaying either the WT or *lht* phenotype were separated and observed under a fluorescence microscope. WT embryos presented newly recruited cells (F), whereas no recruitment was seen in the *lht* mutants (**H**). Scale bar: 50 μm (**A**,**B**). 200 μm (**D**). Each bar represents the mean ± SEM from 4 individual samples. Student’s *t*-test, ***P < 0.0001, N.S., nonsignificant.

Since the chamber specific differentiation of cardiomyocytes was not affected in *lht* mutant hearts, we compared the total number of cardiomyocytes between WT and *lht* mutant hearts. As *lht* mutants lack cardiac looping and distinguishable atrial and ventricular chambers, instead of the number of chamber-specific cells, we counted the total number of cardiomyocytes using MF20/MEF2 staining and endocardial cells using *Tg*(*fli1:GFP*) that expresses GFP in the endocardial cells of the heart. Compared to WT, versican-deficient *lht* mutant embryos displayed 33% fewer cardiomyocytes (Fig. 4A,C), but a similar number of endocardial cells (Fig. 4B,C).

Next, to understand the cause of the reduced number of cardiomyocytes in *lht* mutants, we examined three possibilities: (1) increased apoptosis in the heart of *lht* mutants, (2) reduced cardiomyocyte proliferation in *lht* mutants, and (3) absence of recruitment of progenitor cells from the secondary source in the *lht* mutant heart.

Firstly, we analysed the level of cardiomyocyte apoptosis in live WT and *lht* mutant embryos at 50 hpf using acridine orange staining, but we did not find any apoptotic cells in the heart of the *lht* mutant (see Supplementary Fig. S8). Secondly, using phosphohistone H3 as a cell proliferation marker, we compared the rate of cardiomyocyte proliferation between WT and *lht* mutant embryo at 50 hpf, and found similar rates of cardiomyocyte proliferation in the hearts of WT and *lht* mutant embryos (see Supplementary Fig. S9). Finally, to determine if the progenitor cell derivatives were lost, we examined the expression of elastin b (*elnb*), which marks the SHF-derived smooth muscle of the bulbous arteriosus (BA)^38,39^, by *in situ* hybridization (ISH). In the WT embryos, the expression of *elnb* was prominent in the BA region (Fig. 4D; left panel); in contrast, *elnb* expression was significantly diminished or abolished in versican-deficient *lht* mutant embryos (Fig. 4D; right panel). These results suggested that progenitor cells from the secondary source, from which the BA-specific smooth muscle cells are derived, are absent in *lht* mutants will therefore not be recruited to the arterial pole of the heart tube in *lht* mutants.

Finally, to confirm if recruitment of progenitor cells from the secondary source is absent in the *lht* mutants, we performed a kaede mRNA injection assay^40^. Using UV light (350–400 nm) irradiation, kaede mRNA undergoes irreversible photoconversion from green to red fluorescence. For our experiment, we injected kaede mRNA into a one-cell-stage embryos from an *lht* heterozygous cross and allowed it to develop until 36 hpf, when the linear heart tube forms. Irradiation of the linear heart tube with UV light led to the photoconversion of the kaede protein present in the linear heart tube cells from green to red fluorescence (Fig. 4E,G). At 60 hpf (24 h after irradiation), we observed these embryos under a fluorescence microscope. In the WT embryo, we observed a new population of cells (green fluorescent cell) that migrated in the outflow tract and distal part of the ventricular chamber (Fig. 4F). In contrast, such new cell populations were absent in *lht* mutants (Fig. 4H), suggesting that versican-deficient *lht* mutant embryos lack the recruitment of these progenitor cells to the arterial pole of the heart tube from the secondary source.

Together, these results suggested that versican is important for the migration of these progenitor cells from the secondary source to arterial pole of the heart tube, which contributes to BA development. Also, since the cardiomyocyte count was low, without any significant change in cardiomyocyte proliferation and apoptosis rate, the lack of migration of progenitor cells from the secondary source suggested that these progenitor cells may also contribute to ventricular chamber development.

### The *lht* mutants lack vascular lumen formation

Establishment and stabilization of blood vessels with distinct lumen is vital for vertebrate development. Blood vessels in vertebrates typically develop through two different processes, vasculogenesis and angiogenesis^41^. At the onset of vasculogenesis, endothelial cells develop from angioblast cells and form the vascular cord (primary vascular plexus), and these endothelial cells are closely associated with each other through intercellular tight junctions. The vascular lumen subsequently forms via cord hallowing, in which the endothelial cells migrate from the cord centre to the vessel circumference, with a concomitant reorganization of the tight junctions^42,43^. The yolk sac blood vessels (common cardinal veins; CCV), posterior cardinal veins (PCV), the dorsal aorta (DA), and primordial hindbrain channels (PHBC) constitute the major embryonic vessels that arise during vasculogenesis^44^.

While in angiogenesis, new vessels branch out from pre-existing vessels and the vascular lumen is formed through intracellular vacuole fusion. These nascent blood vessels that develop through both processes are stabilised by the recruitment of smooth muscle cells and ECM secretion^45,46^. Intersegmental vessels (ISVs) are among the first vessels that develop through angiogenesis.

**Figure 5:**
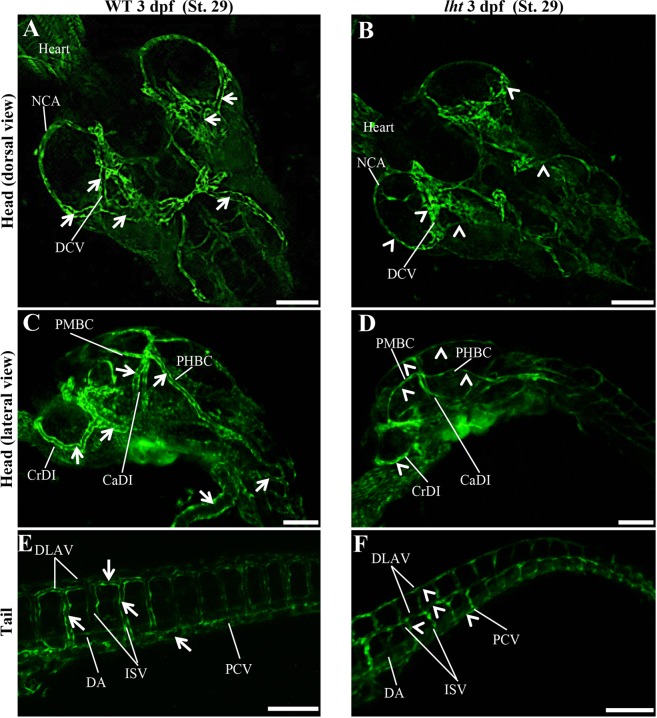
The *lht* mutant shows a complete lack of vascular lumen. Confocal microscope images of the vascular system of WT and *lht* mutant embryos derived from *Tg*(*fli1:GFP*) medaka at 3 dpf (n > 10) (**A**–**F**). White arrows in A, C, and E indicate the vascular lumen. White arrowheads in B, D, and F show the absence of a vascular lumen. BCA, basal communicating artery; CaDI, caudal division of the primitive internal carotid artery; CrDI, cranial division of the primitive internal carotid artery; DA, dorsal aorta; DCV, dorsal ciliary vein; DLAV, dorsal longitudinal anastomotic vessel; ISV, intersegmental vessel; NCA, nasal ciliary artery; PCV, posterior cardinal vein; PHBC, primordial hindbrain channel; PMBC, primordial midbrain channel. Scale bars: 100 μm (**A**–**F**).

Using *Tg*(*fli1:GFP*) transgenic medaka, we found that in WT all the blood vessels that developed either through vasculogenesis (CCV, PCV, DA, and PHBC) or angiogenesis (ISVs) formed lumen (Fig. 5; left panel). But in *lht* mutants, vascular lumen was absent in every blood vessel (Fig. 5; right panel).

To understand the developmental defect in vasculogenesis, we first explored if the *lht* mutants showed any defect in the endothelial migration. Since medaka have large and observable yolk sac blood vessels (common cardinal vein; CCV) connecting heart and embryonic veins, we used CCV as a model to analyse the endothelial behaviour. We performed time-lapse multiphoton laser scanning microscopy using embryos obtained from a heterozygous cross of *Tg*(*fli1:GFP*) transgenic medaka. We found that the endothelial cells migrated in similarly in both WT and *lht* mutants to form a vascular cord (see Supplementary Fig. S10). Further, in WT embryos, the growing endothelial sprouts showed a dynamic extending and retracting pattern, and subsequently developed into a mature blood vessel with a vascular lumen (see Supplementary Fig. S10A–D; Movie 9). However, in *lht* mutants, the endothelial cells sprouted out from the vascular cord and could not retract into the main CCV, resulting in a branched structure without a vascular lumen (see Supplementary Fig. S10E–H; Movie 10).

**Figure 6:**
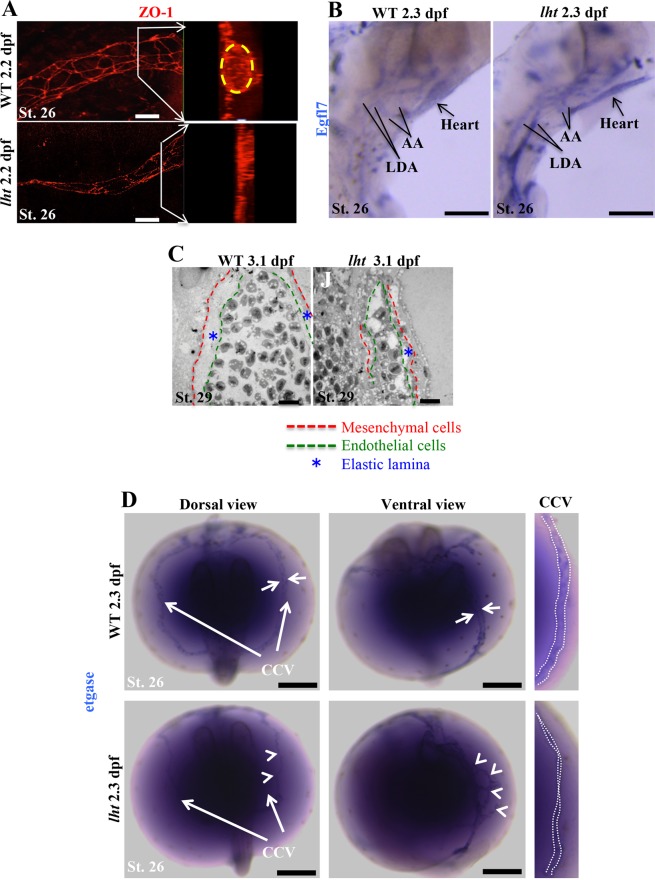
The *lht* mutant embryo exhibits defects in vascular maturation. Whole-mount immunohistochemistry for ZO-1 (a tight junction protein) at 2.2 dpf for WT and *lht* mutant embryos (**A**). Images are 3D projections of z-stack images acquired by confocal microscopy. Inset images display lumen formation in a blood vessel. Yellow circle depicts the vascular lumen in WT embryos (n > 10). *In situ* hybridization for EGFL7 expression in WT and *lht* mutant embryos at 2.3 dpf (n > 10) (**B**). Transmission electron micrograph of a transverse section of the dorsal aorta of WT and *lht* mutant embryos at 3.1 dpf (C; n = 2). *In situ* hybridization for etgase at 2.3 dpf (**D**). White arrows pointing at the CCV in WT represent the wide vascular lumen surrounded by etgase-positive cells. The white arrowheads in *lht* mutant embryos show disoriented etgase-positive cells around a putative blood vessel (n > 10). White dotted lines in the right-most panel indicate the vascular lumen. AA, aortic arch; LDA, lateral dorsal aorta; etgase, embryonic transglutaminase. Scale bars: 20 μm (**A**), 100 μm (**B**), 250 μm (**C**), 10 μm (**B**).

Next, we explored why endothelial cells in *lht* mutant embryos showed unusual patterns after vascular core formation. One possibility of a branched endothelial pattern in *lht* mutants could be the absence of intercellular tight junctions, which are important for endothelial morphogenesis during vascular lumen formation^42,43^. So, we determined if these endothelial cells in *lht* mutants are closely associated via tight junctions. Zonula occludens-1 (ZO-1) is a well-established marker for tight junctions, and localises cell-cell contacts during the early stages of vessel assembly in zebrafish^47^. Therefore, we used a ZO-1 (a tight junction protein) antibody to analyse intercellular tight junction in endothelial cells. The CCV of WT embryos developed a well-organised and elongated endothelial cell network around the periphery of the lumenised vessels (Fig. 6A; Movie 11). However, the CCV of the *lht* mutants showed closely associated endothelial cells with comparable ZO-1 protein expression, although the endothelial cells in *lht* mutants were smaller (Fig. 6A; Movie 12).

Vascular endothelial cells code for a protein, epidermal growth factor-like domain 7 (egfl7) which is associated with ECM during vascular development^48,49^. Egfl7 is expressed at high levels in the vascular bed during embryonic development and is downregulated in most of the mature vessels^50^. It has previously been shown that endothelial cells in egfl7-depleted xenopus embryos remain intact in a vascular cord like structure, fail to undergo cell shape changes, and do not form vascular lumen^47^^,^. However, egfl7 overexpression in mice results in abnormal patterning and remodelling of blood vessels^51^. In addition, egfl7 overexpression causes a subtle hyper-angiogenic response, which results in an elevated number of sprouting endothelial cells^51^. Furthermore, an egfl7 knockdown study in zebrafish suggested that it is important for regulating cord-to-tube transitions^52^. As time lapse images of the developing vasculature in *lht* mutants showed increased endothelial sprouts, we analysed if egfl7 expression was altered in *lht* mutants. Indeed, using *in situ* hybridization, we found high egfl7 expression levels in vascular endothelial cells of *lht* mutants (Fig. 6B). These results suggested that egfl7 expression may have elicited the random migration of endothelial cells.

Besides acting as the core structure of blood vessels, these vascular endothelial cells also synthesise basement membrane, known as elastic lamina^53^. However, they do not appear to be properly deposited without the interaction between endothelial and smooth muscle cells^54^. These smooth muscle cells (mural cells) are differentiated from mesenchymal cells present around the vascular cord^55^ and are recruited into blood vessels during vascular myogenesis^45,46^. Scanning electron micrographs of a transverse section of the dorsal aorta showed that, in WT, the endothelial cell layer is separated from the smooth muscle cellular layer by thick elastic lamina (Fig. 6C; left panel). In contrast, the amount of elastic lamina was very low and, in addition, the endothelial and smooth muscle cell layers were not distinguishable in *lht* mutant medaka (Fig. 6C; right panel).

Embryonic transglutaminase (etgase; ortholog of tissue transglutaminase) is expressed in the mesenchymal cells of regenerating caudal fins of adult medaka. During embryonic development, these etgase-positive cells were exclusively present around the CCVs^56^. Using *in situ* hybridization for etgase, we found that the etgase-positive cells were aligned along the vascular lumen in WT embryos (Fig. 6D; left panel, see Supplementary Fig. S11). However, in *lht* mutant embryos the etgase-positive mesenchymal cells were present around CCV but were disordered (Fig. 6D; right panel).

All these results suggested that although the migration of endothelial cells to form a vascular cord appears to be normal in *lht* mutants, the high expression of egfl7 may have elevated the sprouting pattern of vascular endothelial cells. Further, the alignment of mesenchymal cells along the blood vessels was also disturbed in *lht* mutants. Therefore, *lht* mutant medaka represent a novel genetic model system to study the mechanistic basis of vascular lumen formation in detail.

## Discussion

ECM is an extremely complex and dynamic structure that is continually remodelled to regulate tissue homeostasis. During embryonic cardiovascular development, ECM is enriched in hyaluronan and proteoglycans, including versican. Previous studies using mice have shown that versican repression via a *LacZ* reporter (Vcan ^*hdf/hdf*^)^7,8^, deletion of the hyaluronan binding domain (Vcan ^Δ3*/*Δ*3*^) in a congenic C57BL/6 background^9^, and target deletion of hyaluronan synthase 2 (*Has2*)^10^, resulted in embryonic lethality at day 10.5 of the gestation period owing to severe cardiac defects. However, the *in utero* development of the mouse restricts our understanding of the mechanisms through which ECM molecules regulate cardiac morphogenesis.

Since the cellular and molecular mechanisms underlying cardiac morphogenesis are conserved among vertebrates, teleosts, such as zebrafish and medaka, are ideal organisms to study cardiac development. Using a classic forward genetics approach in the present study, we characterised a newly identified medaka mutant carrying a point mutation in the 3′UTR of the versican gene, resulting in versican loss-of-function. We further confirmed the specificity of versican to *lht* phenotypes using versican morpholino in medaka and zebrafish.

The 3′ (UTR) of mRNA contains regulatory elements that are essential for the appropriate expression of many genes^57,58^. MicroRNAs are small noncoding RNAs that function post-transcriptionally through base pairing to the 3′UTR of mRNA and repress protein synthesis^59,60^. Indeed, the G-A transition in the 3′UTR of the GDF8 allele of Texel sheep created a putative site of miR1 and miR 206, causing translational inhibition of the myostatin gene without affecting mRNA expression^61^. Our preliminary *in silico* results showed that the G-to-T transversion and insertion of thymine residue in *lht* mutants created a putative target site for microRNA-871-3p, which may cause translational inhibition of versican (see Supplementary Fig. S12). However, a future prospective experimental analysis is required to establish the relationship between the mutation site in *lht* mutant medaka and microRNA-871-3p.

The *lht* mutant embryos lacked cardiac jelly and exhibited a constricted outflow tract. Furthermore, heart development in these mutant embryos stopped at the linear heart tube (*lht*) stage owing to a lack of progenitor cells recruitment from the second source, resulting in the absence of BA and lack of a mature ventricular chamber. The strong resemblance between cardiac anomalies in higher vertebrates, such as *hdf* mouse mutant embryos, and lower vertebrates, such as our *lht* medaka mutant, indicated that the role of versican in cardiac development is conserved among vertebrates.

Cardiac precursor cells residing in the splanchnic mesoderm (primary heart field) migrate to a region close to the ventral midline and form a linear heart tube. The linear heart tube then undergoes morphogenesis and develops into a multi-chambered heart by the recruitment of secondary heart field (SHF) and cardiac neural crest cells, collectively called the secondary source of progenitor cells^36,37,62–64^. These cardiac progenitors then localise to the ventricular chamber and outflow tract (also known as bulbous arteriosus; BA in teleosts)^38^. Eventually, the cardiac precursors present in the BA region differentiate into smooth muscle cells through the activity of *elnb*^39^. Previous studies have suggested that versican expression is not obligatory for the formation of the linear heart tube, but is required during the later stages of cardiac morphogenesis^7–9^. However, it is still unclear at which stage the role of versican is most critical, and we investigated this using our *lht* medaka mutant. First, and in line with previous studies^7–9^, we confirmed that versican is not vital until linear heart tube formation since our *lht* mutant embryos were indistinguishable from WT embryos up to this stage. Furthermore, to identify the stage at which versican is essential, our results showed (1) a low number of cardiomyocytes; (2) the absence of recruitment of secondary source progenitor cells towards the arterial pole, using the kaede photoconversion assay; and (3) the absence of *elnb* expression in the *lht* mutant, which suggested that versican expression is critical for facilitating the migration of progenitor cells from the secondary source to the linear heart tube.

The formation of the primary closed circulatory loop requires functional heart and lumenised primary blood vessels. These primary blood vessels are developed through vasculogenesis, and include common cardinal veins CCV), posterior cardinal veins (PCV), the dorsal aorta (DA), and primordial hindbrain channels (PHBC)^44^. During normal vasculogenesis, angioblasts differentiate and form an endothelial network called the vascular cord, which undergoes morphogenesis by recruitment of mural cells derived from splanchnic mesoderm to form mature lumenised blood vessels^65–70^. However, the factors governing these sequential events leading to lumenised vessels are still not fully known.

One of the widely studied factors responsible for vascular lumen formation is hydrodynamic force, *i*.*e*., blood flow. In zebrafish, blood flow is important for shaping and maintaining the vascular lumen through morphological changes in endothelial cell alignment and mural cell recruitment^71,72^. To stop the blood flow, these studies created a silent heart model system either by knocking down the tnnt2 gene, which is required for cardiac function, or by treating embryos with 2,3-butanedione monoxime (BDM), which inhibits cardiac contraction. Interestingly, to determine the effect of hemodynamic forces on vascular lumen formation, intersegmental vessels (ISVs) have been used as a classical model. However, ISVs are sprouted out from the existing vessels via angiogenesis, and are developed after the primary vascular network is established^45,46^. Therefore, it is possible that blood flow may not be necessary for lumen development in primary blood vessels. In fact, primary blood vessels were formed normally in silent heart (*sih*) zebrafish mutants lacking blood circulation^73^. Thus, the establishment of blood circulation may not be prerequisite for the initial establishment of lumen in vessels developed through vasculogenesis. Further studies have shown that endothelial cells can generate lumen independently of blood flow *in vitro*^74^ and during vasculogenesis^47,75,76^. The *lht* medaka mutant exhibited an absence of blood flow and complete lack of vascular lumen in every blood vessel developed either by vasculogenesis or angiogenesis. Therefore, it is possible that the absence of vascular lumen in *lht* medaka mutants is a direct consequence of loss of versican function, but not a secondary effect owing to a lack of blood flow. Future experiments, including the creation of double knockouts of versican and tnnt2a, will provide information about whether loss of versican function affected vascular lumen development in primary blood vessels. For example, if double knockout mutants had silent heart and normal primary vascular patterns in double knock out embryos, then the vascular defect in *lht* mutants is a secondary defect owing to lack of blood flow. However, if double knock out mutants had silent heart and lack of vascular lumen in primary blood vessels, then it could be suggested that versican directly regulates the development of primary blood vessels along with cardiac development.

Moreover, versican is implicated in many biological processes involving vasculature, such as atherosclerosis and vascular inflammation^12–17,77,78^. Recently one study showed that versican directly facilitates tumour growth by promoting angiogenesis, and it was impaired in versican haploinsufficient mice resulting in reduced tumour growth^79^. This study further supports our suggestion that loss of function may have directly affected the vascular lumen formation in *lht* mutants. Future studies using *lht* mutants will help in better understanding the mechanism of vascular development.

In summary, the present study described the cellular and molecular pathology of a novel medaka versican mutant model. This model will provide an opportunity for future investigation into the cellular functions and underlying mechanistic basis of versican during cardiovascular development. Furthermore, our model system is suitable to study the relationships between flow dynamics and vascular lumen formation.

## Materials and Methods

### Fish maintenance and genetic screening

Wild-type Qurt and HNI medaka (*O*. *latipes*) strains were raised and maintained at 28.5 °C in a recirculating water system under a reproduction photoperiod (14 h light/10 h dark). ENU was used for mutagenesis, and a standard genetic F3 screen for mutations affecting embryogenesis was performed as previously described^21^. The *lht* mutant embryos were isolated by microscopic inspection as a Mendelian-inherited recessive lethal mutation that results in the absence of cardiac looping and blood flow. The collected fertilised eggs were incubated at 28.5 °C in medaka Ringer′s solution (0.65% NaCl, 0.04% KCl, 0.011% CaCl_2_, 0.01% MgSO_4_, 0.01% NaHCO_3_, and 0.0001% methylene blue), and the developmental stages were determined by morphology according to the medaka staging table. The *Tg*(*fli1:GFP*) transgenic line was kindly provided by Dr. M. Furutani-Seiki (University of Bath, Bath, UK).

### Adult medaka euthanisation and organ collection

All procedures were conducted in accordance with the Laboratory Animal Welfare Act and the Guide for the Care and Use of Laboratory Animals (National Institutes of Health, Bethesda, MD, USA). All experiments were approved by the local Animal Experiment Committee of CIEA of Japan and the Animal Care and Use Committee of Keio University (Registration Number: 4791). Adult medaka were euthanised using diluted ethyl 3-aminobenzoate methanesulfonate salt (Tricaine, MS-222: Sigma-Aldrich, St. Louis, MO, USA). Stock solution of Tricaine (4 g/L; pH7) was diluted to 300 ug/ml. The adult medaka remained in the solution for 10 min following cessation of opercular (gill) movement. The zebrafish were anaesthetised with ice water. Within 15 min the ovaries, testes, eyes, brain, gills, and heart were dissected as prescribed previously^80^. The skeletal muscle was dissected as per protocol described here^81^. Total RNA was isolated from organs using the RNeasy Mini kit (Qiagen) and treated with DNase.

### Histological analysis

Medaka embryos were fixed with 4% paraformaldehyde (PFA) in phosphate-buffered saline (PBS) overnight at 4 °C, dehydrated in ethanol, embedded in paraffin wax, sliced into 5-μm sections, and stained with H&E. The stained sections were imaged using a BIOREVO optical microscope (BZ-9000; Keyence, Osaka, Japan).

### Transmission electron microscopy

Samples were prepared as described previously^82^, sectioned using an RMC MT6000 ultramicrotome, examined under a transmission electron microscope JEM-1230 (JEOL, Tokyo, Japan), and photographed using Digital Micrograph 3.3 (Gatan Inc., Pleasanton, CA, USA).

### Genetic mapping and positional cloning

Genetic mapping was performed according to the method described earlier^83^. Briefly, a *lht* mutant in the Qurt genetic background was crossed with an HNI strain to obtain the genetically polymorphic Fl offspring. Fl were in-crossed to obtain *lht* mutant embryos for mapping. Pooled bulked segregant analysis was performed to locate the *lht* mutation on chromosome 9 of medaka^84^. Further mapping was performed using custom-designed markers based on the sequence polymorphism between the Qurt and HNI strains. The mapping primers and restriction enzymes used for mapping are given in Supplementary Table 1.

### Extraction of genomic DNA and sequence analysis

Genomic DNA was extracted from the fins of adults or from the whole larvae using a REDExtract-N-Amp™ Tissue PCR kit (Sigma, St Louis, MO, USA) according to the manufacturer′s instructions. DNA integrity was confirmed by gel electrophoresis. In addition, 2 μL of purified DNA was amplified using a Taq DNA polymerase kit (TaKaRa, Shiga, Japan). Both sense and antisense strands of all the amplicons were sequenced using the BigDye™ Terminator v3.1 cycle sequencing kit (Applied Biosystems, Foster City, CA, USA) and ABI PRISM™ 3130xL Genetic Analyzer (Applied Biosystems). Medaka were genotyped using following set of primers:

Forward Primer: Olvcan 3′UTR-F1: 5′-TTCAGGCTATAGGCGCTACC-3′

Reverse Primer: Olvcan 3′UTR-R1: 5′-CAAACGTTGGGTCCAGTTCT-3′

### RNA extraction and RT-PCR

Total RNA was isolated from whole embryos and isolated hearts at various stages of development using TRIzol™ (Invitrogen, Carlsbad, CA, USA) following the manufacturer’s specifications and treated with amplification-grade DNase I (1 U/μg RNA; Invitrogen). SuperScript III RNase H-reverse transcriptase (Invitrogen) was used for first-strand cDNA synthesis, using oligo(dT) primers and 1 μg total RNA for 50 min at 50 °C. To evaluate mRNA splicing, primers were designed for exon boundaries as indicated in Supplementary Table 2. PCR amplification was carried out using KOD-FX (Toyobo, Tokyo, Japan) DNA polymerase.

### q-PCR analysis

Medaka embryonic heart and cells were used for total RNA isolation. cDNA was synthesised using 1 μg total RNA and a SuperScript® III kit (Thermo Fisher Scientific, Waltham, MA, USA). q-PCR was performed using ViiATM7 (Applied Bio, Waltham, MA, USA) and SYBR® Green PCR Master Mix (Applied Bio, Waltham, MA, USA). The primer pairs are listed in Supplementary Table 3.

### Microangiography

Microangiography was performed as previously described^85^. In brief, green fluorescent microspheres (diameter, 0.02 pm) (Molecular Probes, Eugene, OR, USA) were diluted 1:1 with a 2% bovine serum albumin (Sigma, St Louis, MO, USA) solution and then sonicated. WT and *lht* embryos were anesthetised in tricaine as previously described^86^ before being mounted ventral side up in 1% (w/v) low-melting agarose (Sigma) in E3 medium. The microsphere suspension was then injected either into the sinus venosus (for 2-dpf embryos) or directly into the heart (for 6-dpf embryos) using a pressure injection system (Narishige, Amityville, NY, USA). The efficiency of injection was monitored under an MZFLIII stereo-microscope equipped with a GFP filter set (Leica, Wetzlar, Germany).

### Whole-mount *in situ* hybridization

Whole-mount *in situ* hybridization was performed according to a previously described procedure^87^. After staining, tissues were fixed with 4% PFA in PBS for colour preservation, equilibrated with 80% glycerol, and mounted on slide glasses for microscopic observation. The primers used for cloning the respective probes by PCR are mentioned in Supplementary Table 4. The PCR products were cloned using Zero Blunt® TOPO® PCR Cloning Kit (Invitrogen) according to the manufacturer’s protocol and validated by sequencing. Sense and antisense riboprobes were generated with DIG RNA Labelling Mix (Roche Diagnostic, Indianapolis, IN. US) by *in vitro* transcription using T7 polymerase (Roche-Diagnostic) and T3 polymerase (Roche-Diagnostic) with plasmids linearised with *Eco*RI and *Not*I.

### Immunohistochemistry and cell count assay

We performed whole-mount immunohistochemistry using an anti-MF20 (Developmental Studies of Hybridoma Bank) antibody, anti-MEF2 (sc-313; Santa Cruz Biotechnology, Santa Cruz, CA, USA) antibody to count cardiomyocytes at various stages of development, and Phospho-Histone H3 (Ser10) Antibody  (9701, Cell Signaling Technology, Danvers, MA) to count proliferating cardiomyocytes. The embryos were grown to the desired developmental stage and subsequently fixed overnight at 4 °C in 4% PFA and washed with PBS containing 0.1% Tween 20 the following day. Immunofluorescence was performed as described previously^88^. The embryos were flat-mounted in 50% glycerol and imaged ventrally using a Carl Zeiss confocal microscope LSM 510 META, with a 20-μm lens for the heart tube overview and a 60-μm immersion lens for cell count of the ventricle and atrium. Sequential confocal images were taken with excitation at 488 and 568 nm, and a standardised z-stack size of 0.642 μm. Three-dimensional (3D) reconstructions of confocal stacks were made using 3D projection software (Carl Zeiss). The MEF2- and MF20-positive cells were confirmed on each z-stack. All embryos were counted at least three times.

### Combined *in situ* hybridization and immunohistochemistry

Following *in situ* hybridization, immunohistochemistry was performed using anti-MEF2 and anti-MF20 antibodies conjugated with alkaline phosphatase. After Fast Red (Roche, Basel, Switzerland) application, Alexa 488 and 610 secondary antibodies were applied for anti-MEF2 and anti-MF20 antibodies, respectively.

### Apoptosis cell count assay

To count apoptotic cells, WT and mutant embryos were mounted in a 2 µg/mL solution of acridine orange (Sigma) in PBS for 30 min at room temperature. After washing several times with PBS, apoptotic cells were observed with excitation at 488 nm using an LSM 510 laser scanning microscope (Zeiss).

### Morpholino (MO) injections and control experiments

Versican-targeting morpholino (Gene Tools) was dissolved in 1× Yamamoto ringer solution. Embryos were injected with different dilutions of MO at the one-cell stage. The versican-targeting MO (VCAN MO: 5′-GTAAGTCCTTAAACTGAATGAGTCC-3′) and a standard negative control oligo (N.Ctrl MO: 5′-CCTCTTACCTCAGTTACAATTTATA-3′), were injected at 0.1 and 0.5 mM.

### Western blotting

Cellular lysates and IPs were separated by SDS-PAGE and transferred to polyvinylidene fluoride (PVDF) membranes. After preincubation with 3% skimmed milk in Tris-buffered saline (TBS), the membrane was incubated overnight with the anti-Versican/PG-M mAb primary antibody, clone 4D1 (MC-955, Kamiya Biomedical Company, Seattle, USA). After washing with TBS, the membrane was incubated with the appropriate secondary antibody (anti-rabbit IgG horseradish peroxidase-conjugated Ab, 1:10000, in 3% skimmed milk in TBS; GE Healthcare) for 1 h. After five washes with TBS with 0.1% Tween 20, signals were visualised using Chemi-Lumi One Super (Nacalai) and Luminescent Image Analyzer LAS-3000 mini (Fujifilm, Tokyo, Japan).

### Photoconversion of kaede fluorescence

Photoconversion of kaede fluorescence from green to red was achieved by exposing the mutant and WT embryos to UV light on a Zeiss Axioplan microscope equipped with a DAPI filter set, as previously described^40^. Confocal z-stacks were obtained using an LSM 510 laser-scanning microscope (Zeiss) and analysed with Zeiss LSM and Velocity software.

### Time-lapse microscopy

*Tg*(*fli1:GFP*) embryos from a heterozygous cross were dechorionated using a previously described method^89^. The embryo medium was prepared with tricaine (0.016%) to inhibit embryo movement. At 36 hpf, embryos were embedded in 0.8% low-melting agarose in a glass bottom 35 mm Petri dish. The Petri dish was sealed with a Teflon membrane, which allows air to flow through while rendering it impermeable to liquids, preventing embryos from drying out during imaging. The dish was kept in a water-jacketed incubator maintained at 28 °C, and time-lapse recording was performed using an inverted confocal laser scanning microscope (LSM 510 META; Zeiss) with minimal laser excitation to prevent photodamage and photobleaching. Z-stacks were collected every 20 min for 14 h using a 10× objective lens with 45% zoom. The z-stacks were combined into a single projection and exported as a movie using LSM image browser (Zeiss) software with 4 frames per second.

### Establishment of medaka cell lines and immunocytochemistry

Medaka cell lines from 2-dpf WT and *lht* mutant embryos were established according to a previously described procedure^90^. Here, we used 33 °C as the incubation temperature for maintenance. For immunocytochemistry, 2 × 10^8^ cells were seeded on 35 mm glass bottom dishes and incubated for 4 d. After incubation, the cells were washed with PBS, fixed with 4% PFA, and permeabilised using 0.1% Triton X-100. The cells were incubated with anti-Versican/PG-M mAb primary antibody, clone 4D1 (MC-955, Kamiya biomedical company, Seattle, USA) for 3 h at room temperature. The cells were then incubated with Goat Anti-Mouse IgG H&L (Alexa Fluor® 488, ab150113, Life Technologies, Carlsbad, CA) and DAPI for 1 h at room temperature.

### Delivery of morpholino oligos with nucleofection

The delivery of morpholino oligos with nucleofection was performed according to a previously described procedure^91,92^. Briefly, for the nucleofection of medaka fibroblasts, cells isolated from WT embryos were grown to approximately 80% confluency (generally such confluency is achieved 36 h after seeding) in L15 media with 15% FBS. The cells were harvested using TripLE and spun down. 3 × 10^6^ cells were resuspended in 100 μl T solution (Amaxa Inc., Gaithersburg, MD, USA) and mixed with 2 μM control MO or versican MO. The mixture was then transferred into a kit-provided cuvette and the cells were electroporated using a nucleofector device (Amaxa Inc., Gaithersburg, MD, USA) and program O-20, as per manufacturer’s instructions. Immediately after electroporation, 500 μl of the prewarmed (33 °C) culture medium were added into the cuvette, and then transferred into a culture medium in collagen I treated 35 mm glass bottom dish. After 24 h, the dishes were washed to remove non-adhered cells and new medium was added for further incubation. After 72 h of incubation at 33 °C, an immunocytochemistry procedure was performed to analyse versican expression using anti-Versican/PG-M mAb primary antibody, clone 4D1 (MC-955, Kamiya biomedical company, Seattle, USA).

### Statistical analysis

Data of all samples were compared using the independent. t-test with equal variances. Results were considered statistically significant at a value of *p < 0.05, **p < 0.01 for all comparisons.

## Supplementary information

Combined supplementary file

Dataset 1

Movie 1: Representative video of live imaging of cardiovascular system of a 50 hpf WT embryo.

Movie 2: Representative video of live imaging of cardiovascular system of a 85 hpf WT embryo.

Movie 3: Representative video of live imaging of endocardial development of a 60 hpf WT embryo, using Tg(fli1:GFP)

Movie 4: Representative video of live imaging of cardiovascular system of a 50 hpf lht mutant embryo.

Movie 5: Representative video of live imaging of cardiovascular system of a 85 hpf lht mutant embryo.

Movie 6: Representative video of live imaging of endocardial development of a 60 hpf lht embryo, using Tg(fli1:GFP)

Movie 7: Live imaging of versican morpholino injected medaka embryo, showing the effect on cardiovascular development.

Movie 8: Live imaging of versican-a morpholino injected zebrafish embryo, showing the defect on cardiovascular development.

Movie 9: Time-lapse imaging of vascular lumenization using Tg(fli1:GFP) in WT.

Movie 10: Time-lapse imaging of vascular lumenization using Tg(fli1:GFP) in lht mutant.

Movie 11: 3D reconstruction from confocal Z‐stack images of whole mount immuno-histochemistry of adherens junction related molecule ZO-1 in CCV of WT embryo.

Movie 12: 3D reconstruction from confocal Z‐stack images of whole mount immunohistochemistry of adherens junction related molecule ZO-1 in CCV of lht mutant embryo.

Movie 13: Movement response of WT embryo against needle touch.

Movie 14: Movement response of lht embryo against needle touch

## Data Availability

The datasets generated and analysed during the current study are available from the corresponding author on reasonable request.
