# Supplementary material for: Versican is crucial for the initiation of cardiovascular lumen development in medaka (Oryzias latipes)
Source: Sci Rep. 2019 Jul 1;9:9475. doi: 10.1038/s41598-019-45851-3 (PMC6603046; doi:10.1038/s41598-019-45851-3)
Supplement: Supplementary file 1 — Combined supplementary file [file 41598_2019_45851_MOESM1_ESM.pdf]

Versican is crucial for the initiation of cardiovascular lumen development in medaka (*Oryzias latipes*)

Nishant Mittal[1]; Sung Han Yoon[2]; Hirokazu Enomoto[1]; Miyama Hiroshi[1]; Atsushi Shimizu[3]; Atsushi Kawakami[4]; Misato Fujita[5]; Hideto Watanabe[6]; Keiichi Fukuda[1]; Shinji Makino[1,7]*

[1] Department of Cardiology, Keio University School of Medicine, 35-Shinanomachi Shinjuku-ku, Tokyo 160-8582, Japan

[2] Department of Interventional Cardiology, Cedars-Sinai Medical Center, 8700 Beverly Boulevard, AHSP A9229, Los Angeles, CA 90048, USA

[3] Division of Biomedical Information Analysis, Iwate Tohoku Medical Megabank Organization, Iwate Medical University, 2-1-1 Nishitokuta, Yahaba-cho, Shiwa-gun, Iwate 028-3694, Japan

[4] Department of Biological Information, Tokyo Institute of Technology, 4259 Nagatsuta, Midori-ku, Yokohama 226-8501, Japan

[5] Department of Biological Science, Graduate School of Science, Kanagawa University, 2946 Tsuchiya, Hiratsuka-Shi, Kanagawa-Ken 259-1293, Japan

[6] Institute for Molecular Science of Medicine, Aichi Medical University, 1-, Yazakokarimata, Nagakute, Aichi 480-1195, Japan

[7] Keio University Health Centre, 35-Shinanomachi Shinjuku-ku, Tokyo 160-8582, Japan

*** Corresponding author:** **smakino@keio.jp**

Supplementary figures

Fig. S1: Wild-type and *lht* mutant embryos are phenotypically indistinguishable. Phase contrast microscopic image showing representative WT and *lht* mutant embryos at 40 hpf (n = 10). Wild-type and mutant embryos were phenotypically similar at this stage. Scale bar: 0.2 mm (A–D). Heartbeat rate of WT and *lht* mutant medaka embryo, at 2 dpf and 3 dpf (n = 13). The WT and *lht* mutant medaka exhibited a similar heartbeat rate (E,F). Each bar represents the mean ± SEM from 13 individual samples. Student's *t*-test, ***P < 0.0001, N.S., not significant.

Fig. S2: Amino acid sequence alignment of medaka, human, and mouse versican. Amino acid sequence of medaka, human, and mouse versican aligned by the ClustalW program shown with their UniProt accession codes. The black box indicates the region of high homology and corresponds to the G1 and G3 domains. The rest of the amino acid sequence belongs to the G2 domain.

Fig. S3: Analysis of versican mRNA expression in medaka. RT-PCR and qPCR data for versican expression in various organs of adult medaka (A, B). Versican expression was prominent in the heart, eyes, gills, muscle, ovary, and testis. Whole-mount *in situ* hybridization for versican at multiple stages of embryonic development (C). Versican expression was prominent in the eye, heart tube, and ICM until 48 hpf (C; left and middle panels). At 72 hpf, the embryo shows strong versican expression in the gill region (C; right panel). Versican expression in the heart at various stages of cardiac development (D). (n > 10) (B). ICM, intermediate cell mass. Scale bar: 500 μm (C), 100 μm (D). Each error bar represents the SEM from 3 individual samples. β-Actin was used as endogenous reference gene to calculate ΔCT value. To calculate ΔΔCT, testis was selected randomly as reference sample.

Fig. S4: The 3' UTR sequence of the versican region of interest is conserved in all medaka strains. We used the same set of primers we designed for genotyping, and analysed the sequence for QURT1, HN1, Hd-rR, Kaga, and Ok-cab. The region of interest did not show any polymorphism (A). Western blot results for the versican expression in WT embryo, *lht* embryo, WT cell line transfected with either ctrl MO or versican MO (B). This result was obtained when a new lot of MC-955 (versican) antibody was used. Cell lines derived from WT medaka embryos were transfected with either ctrl or versican MO, and immunoassayed with versican G2 domain specific (MC-955) antibody (C). From three independent experiments, the total number of cells expressing versican around the cell boundary were counted using GFP

expression and nuclear staining by DAPI (D). Morpholino (MO) injection assays and quantification data (E–F). Versican-a MO was injected at the one-cell stage into embryos obtained from WT zebrafish. A morphant injected with versican-a MO (0.5 mM) phenocopies the *lht* mutant (C). Graphical representation of versican-a MO (0.5 mM) showing all the *lht* mutant phenotypes (D) ctrl: control. The results are represented as mean percentages of three independent experiment, mean ± SD.

Fig. S5: Comparative study of mRNA and protein levels in WT and *lht* mutant medaka. Structural representation of the V0, V1, V2, and V3 versican isoforms (A). Versican mRNA expression data for WT and mutant embryos using qPCR (B). RT-PCR results show the comparison of mRNA expression levels at the exon-exon boundary of WT and the *lht* mutant (C). Primers were designed for exon boundaries. Coloured regions indicate the specific isoform exon-exon boundaries. RT-PCR results show the comparison of mRNA expression levels at the exon-exon boundaries of WT and the *lht* mutant (n = 3). Cell lines derived from WT and *lht* mutant embryos were immunoassayed with a versican G1 domain-specific antibody. In the WT-derived cell line, versican was expressed at the cell membrane (D; left panel). However, versican expression was absent at the cell membrane of the *lht* mutant-derived cell line (D; right panel). Each error bar represents the SEM from three individual samples. β-Actin was used as endogenous reference gene to calculate ΔCT value. To calculate ΔΔCT, WT was selected as reference sample. Student's *t*-test, $^{***}P < 0.0001$, N.S., not significant.

Fig. S6: The *lht* mutant displays constriction of the outflow tract. Stereomicroscope images of embryos injected with fluorescence beads into the sinus venosus at 4 dpf. Panels A and B show the bright field images and panels C and D show the fluorescent images. The fluorescent beads injected into the heart (C) flowed into the vascular network of WT embryos (D), but pooled in the heart tube of *lht* mutant embryos that lacked blood circulation. Scale bar: 200 μm (A–D)

Fig. S7: Versican loss of function did not alter the chamber-specific differentiation of cardiomyocytes. *In situ* hybridization for the cardiac-specific genes *cmlc2* (A, B), *amhc* (C), and *vmhc* (D) (n > 10) in the heart at 3 dpf. Red circle marks the presence of *cmlc2*-positive cells localized near the outflow tract. Scale bar: 100 μm (A–D).

Fig. S8: The heart of the *lht* mutant medaka embryo did not show any apoptosis at 50 hpf. Analysis of apoptosis in live embryos with acridine orange staining examined with a

KEYENCE fluorescence microscope. We did not find any apoptotic cells in the *lht* mutant heart. The white arrow indicates acridine orange positive apoptotic cells in the head region of WT and *lht* mutants.

Fig. S9: The heart of the *lht* mutant medaka embryo did not show any differences in cardiomyocyte proliferation compared to that of WT at 50 hpf. Analysis of cardiomyocyte proliferation by whole-mount immunohistochemistry at 50 hpf, using a phosphohistone H3 antibody. The z stack images were obtained by confocal microscopy (A, B). There was no significant difference in the rate of cardiomyocyte proliferation between WT and *lht* mutant embryos (C). White arrows indicate the phosphohistone H3 positive cells in the heart. Each bar represents the mean ± SEM from nine individual samples. Student's *t*-test, ***$P < 0.0001$, N.S., not significant.

Fig. S10: The *lht* mutant shows disrupted endothelial alignment. Time-lapse microscopic images of WT (n > 15) (A–D) and *lht* mutant embryos (n = 5) (E–H) at 38, 42, 46, and 50 hpf. Embryos obtained from a Tg(fli1:GFP):*lht* heterozygous cross were dechorionated and embedded in 1% low-melt agarose. These embryos were kept under the microscope for the duration of the time-lapse imaging from 32 to 50 hpf. Images were taken at 20 min intervals. The temperature was maintained at 28.5°C throughout the experiment. A, atrium; CCV, common cardinal vein; V, ventricle. Scale bar: 100 µm.

Fig. S11: The expression of etgase is specific to yolk sac blood vessels. Bright field image of etgase mRNA expression in WT embryos, after removal of the yolk, using *in-situ* hybridization. etgase was expressed exclusively in yolk sac blood vessels.

Fig. S12: Mutation in 3'UTR in the *lht* mutants creates putative binding sites for microRNA mmu-miR 871-3p.

Fig. S13: Full-length gels of Figure 2F. Note: WT: wild type, het: heterozygous, *lht: lht* homozygous mutant.

Fig. S14: Full-length gels of Figure S4B. Note: WT: wild type, *lht: lht* homozygous mutant, Ctrl MO: medaka fibroblasts nucleofected with control morpholino, versican MO: medaka fibroblasts nucleofected with versican morpholino.

Fig. S1

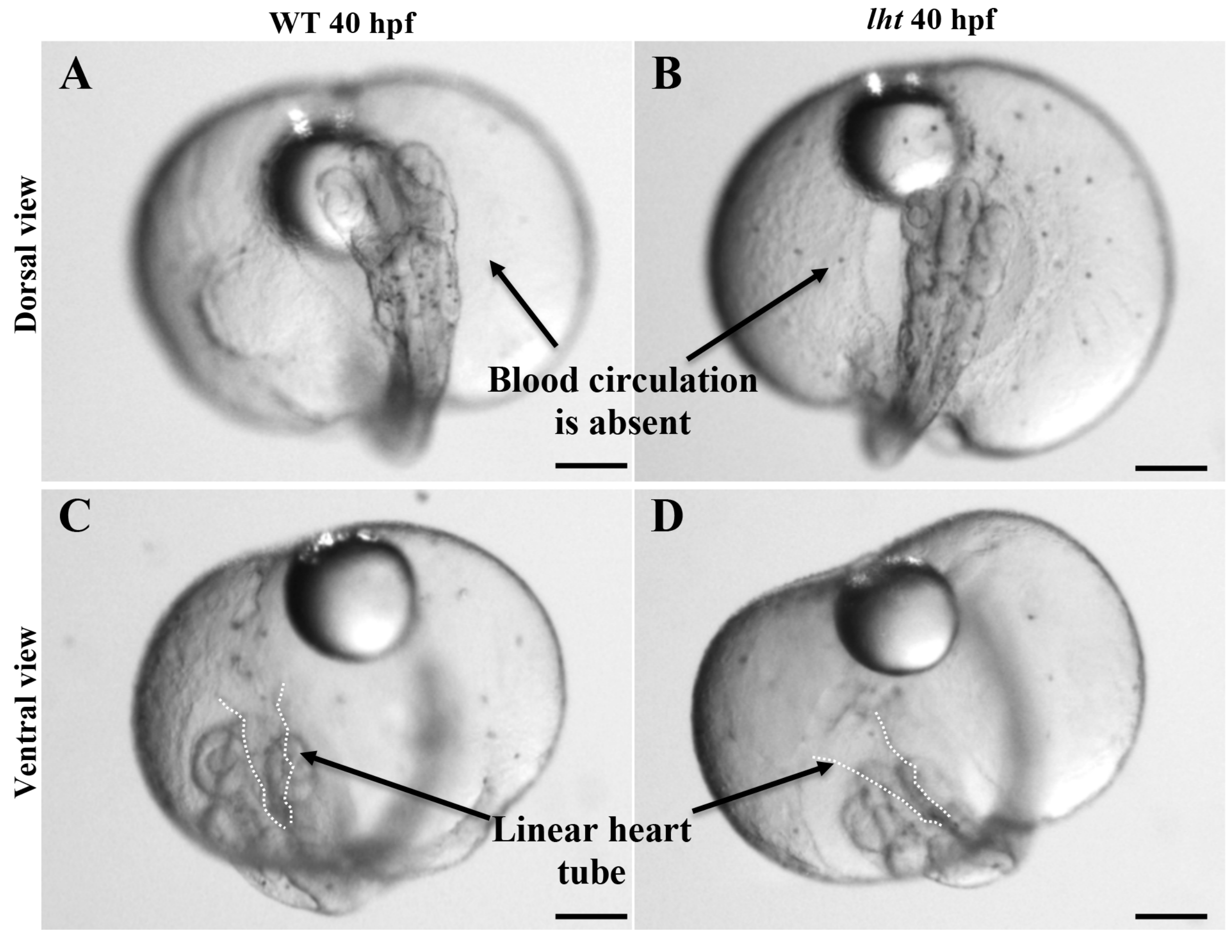

E

Embryo stage	WT embryo (Mean ± SEM)	*lht* mutant embryo (Mean ± SEM)	P value
2 dpf	109 ± 2.14	105 ± 1.66	0.145
3 dpf	125.92 ± 1.51	125.77 ± 1.87	0.956

F

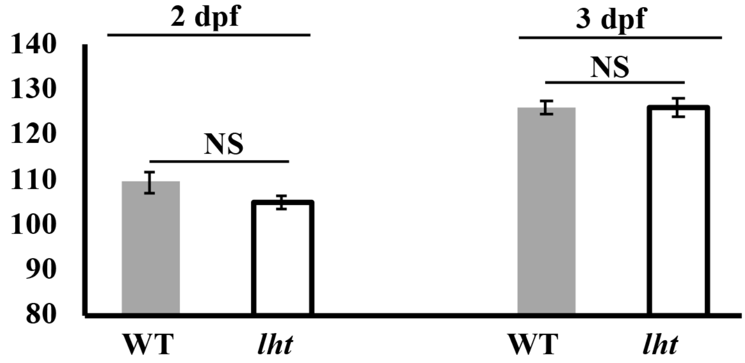

G1 domain

```
MEDAKA MILNIRHILWLYYLSEAAAATASHAISILKPITGSLSGKVNLPCYFSTIPTSAPLIGPNG 60
HUMAN  MFINIKSILWMCSTLIVTHALHKVKVGKSPPVRGSLSGKVSLPCHFSTMPTLPPSYN--- 57
MOUSE  MLINMKGILWMCSTLLLTHALHQAKMETSPPVKGSLSGKVVLPCHFSTLPTLPPNYN--- 57
       *::*:: ***:      : *  .  :    *: ******* ***:***:**  *  .

MEDAKA SITYGRDLLRIKWTKIQRN------TESVVLVAQNGVIKIGKSYRNRVSVPSHPEDVGDA 114
HUMAN  ----TSEFLRIKWSKIEVDKNGKDLKETTVLVAQNGNIKIGQDYKGRVSVPTHPEAVGDA 113
MOUSE  ----TSEFLRIKWSKMEVDKNGKDIKETTVLVAQNGNIKIGQDYKGRVSVPTHPDDVGDA 113
             ::*****:*::  :     .*:.******* ****:.*:.*****:**: ****

MEDAKA SLTMVKLRASDAGTYRCEVLYGIEDTQDTISLDVRGVVFHYRAKTSRYTLDYQKAVRACQ 174
HUMAN  SLTVVKLLASDAGLYRCDVMYGIEDTQDTVSLTVDGVVFHYRAATSRYTLNFEAAQKACL 173
MOUSE  SLTMVKLRASDAGVYRCDVMYGIEDTQDTMSLAVDGVVFHYRAATSRYTLNFAAAQQACL 173
       ***:*** ***** ***:*:*********:** * ******** ******::  * :**

MEDAKA DIGATIATYDQLKAAYEDGFDQCDAGWIADQTVRYPITKPRKGCFGNLMTNPGIRSYGTR 234
HUMAN  DVGAVIATPEQLFAAYEDGFEQCDAGWLADQTVRYPIRAPRVGCYGDKMGKAGVRTYGFR 233
MOUSE  DIGAVIASPEQLFAAYEDGFEQCDAGWLSDQTVRYPIRAPREGCYGDMMGKEGVRTYGFR 233
       *:**.**: :** *******:******::********  ** **:*: * : *:*:** *

MEDAKA KPTETYDVYCYVDKLDGEVYYAPVTRMMTFEEASEECKKRNAVLATPGQLHAAWRQGLDR 294
HUMAN  SPQETYDVYCYVDHLDGDVFHLTVPSKFTFEEAAKECENQDARLATVGELQAAWRNGFDQ 293
MOUSE  SPQETYDVYCYVDHLDGDVFHITAPSKFTFEEAEAECTSRDARLATVGELQAAWRNGFDQ 293
       .* **********:***:*::  .   :*****  ** .::* *** *:*:****:*:*:

MEDAKA CDYGWLSDGSVRHPVAVSRIQCGGGLLGVRTMYRYRNQTGFPEPTMKLGAYCFKGRRDVF 354
HUMAN  CDYGWLSDASVRHPVTVARAQCGGGLLGVRTLYRFENQTGFPPPDSRFDAYCFKPKE--- 350
MOUSE  CDYGWLSDASVRHPVTVARAQCGGGLLGVRTLYRFENQTCFPLPDSRFDAYCFKPKQNIS 353
       ********.******:*:* ***********:**:.*** ** *  ::.***** :.

MEDAKA NQTSLVDVSVIDITTASIVSTTSMPLLESSTNAVSTTSAPVTEGDQSKDSAAATNTPFMF 414
HUMAN  -ATTI-DLSIL--------AETASPSLSKEPQMVSDRTTPI--------------IPLVD 386
MOUSE  EATTI-EMNIL--------AETSSPSLSKEPHMVPDRATPV--------------IPLAT 390
         *:: ::.::        : *: * *... : *   ::*:                *:

MEDAKA STSMAPSSFPSADREEDLIT--TIAPTIKEEDDTFDI------VTPMFNIDDFGTVDEES 466
HUMAN  ELPVIPTEFPPVGNIVSFEQKATVQPQAITDSLATKLPTPTGSTKKPWDMDDYSPS---- 442
MOUSE  ELPIFTTHFPPAGNIVNSEQKSVVYSQAITGRLAMESPTTTRNTINSWDLNDSLAS---- 446
       .  :  : ** ...  .     .:         : .       .   ::::*

MEDAKA VPKRGDVVRYPSIPTESSNDTVSDPDDPSVIKISTIQPDVLMPGSSVDTKT-MFAEGKTE 525
HUMAN  ------------------------ASGPLGKLDISEIKEEVLQSTTGVSHYATDSWDGVVE 479
MOUSE  ------------------------GSGPLGMPDISEIKEEELRSTTVISQHATGSQAVITE 483
                                .  .  .** *: : *   : :.  :       .*

MEDAKA EAFVHSSITAAVTSDVTETPKGAIPEETTPFPDYDKDIDDTFIHVEVMPPVQ---VFNEE 582
HUMAN  DKQTQESVTQI--------------------------------EQIEVGPLVTSMEILKHI 508
MOUSE  DTQTHESVSQI--------------------------------EQIEVGPLVTSMEITNHI 512
       :  .:.*::                                  ::** * *    : :.

MEDAKA PSS-------SPATST-TTVTTPLMCKTLPGTTELPSTTHTGTQTILETHDMDSNAKSGT 634
HUMAN  PSKEFPVTE-TPLVTARMILESKTEKKMVSTVSELVTTGHYGFTL-GEEDDEDRTLTVGS 566
MOUSE  SLKELPEKNKTPYESTEVTLEHTTEMPTVSASPELATTSHYGFTL-REDDREDRTLTVRS 571
         .       :*  ::   :        :    ** :* * *     * .  * . .  :

MEDAKA TAITKVDGETTTPHETLSARGDIFDESGSQTAVQKTGTEAEG----QL---FTSTRTAPA 687
HUMAN  D-----------------ESTLIFDQIPEVITVSKTSEDTIHTHLEDLESVSASTTVSP- 608
MOUSE  D-----------------QSTRVFSQIPEVITVSKTSEDTTYSQLGDLESISTST----- 609
                             :*.:  .  :*.**. ::      :*    :**
```

```
MEDAKA AASVLTSRSAVISEFQPL-ETSDITKTQDASGNPPHTVPLNL*IEQLK*QMLLLDFC*FN 746
HUMAN  -LIMPDNNGSSMDDWEERQTSGRITE--EFLGKYLSTTPF---PSQHRTEIELFPYSGDK 662
MOUSE  -ITMLGTDRSLIDKEKEPKTNGKVTE--DEFGQSQPTTTF---PSQHLTEVELLPYSGDT 663
          :  .  : :.. :    .. :*:  :  *:   *. :    .*   :: *: :.  .

MEDAKA LI*FMFGIHLVYFLEI*ITVIMVLLPQWLL*VKA*VS*RFVSL*VLFLRILVY--DEV-- 802
HUMAN  --ILVEGISTVIYPSLQT------------EMTHRRE-RTET-LIPEMRTDTYT-DEIQE 705
MOUSE  --TSVEGISTVIYPSLQT------------DVTQGRE-RTET-PRPELKKDPYTVDEIPE 707
           : **  * : .:               :.   . *  :     ::   *  **:

MEDAKA ------WLQGS*TSYYN*FQIGEKRS--FLCI--KKEFTKNNRFFLFFLLFLVCLYVFSI 852
HUMAN  EITKSPFMGKTEEEVFSGMKLSTSLSEPIHVTESSVEMTKS---FDF-----------PT 751
MOUSE  KVTKDPFIGKTE-EVFSGMPLSTSSSES------SVERTES---VSP-----------AL 746
             ::  :  . :. : :. . *        . * *:.   .

MEDAKA YLLQVFFK*SDILILT----ERR*V*NAAH*YSEIT*M*LFHSQPCSFLIIWGFKIVLEV 908
HUMAN  LITKLSAEPTEVRDMEEDFTA---TPGTTKYDENITTVLLAHGT---------------- 792
MOUSE  TIEKLTVKPTEARDVEEMTTLTRLETDVTKSDKDVTRVHLTHST---------------- 790
        : ::  : ::   :           ..::  .::* : * *.

MEDAKA TFF*PICFKVYLKKILFMLV*SMINFSIMFCCSFS*H*NVMFQLFCFFSASSV*VFFWHT 968
HUMAN  -----------------------LS-----------------------VEAATVSKWSW 805
MOUSE  -----------------------LN-----------------------VEVVTVSKWPG 803
                              :.                        .. . *  *

MEDAKA T*IKFC*KQLS*----------QVLYFNIFIN--FKENPSL*SRTG-------------V 1003
HUMAN  DEDNTTSKPLESTEPSASSKLPPALLTTVGMNGKDKDIPSFTEDGADEFTLIPDSTQKQL 865
MOUSE  DEDNSTSKPLPSTEHAGFTKLPPVPLSTIGINGKDKEIPSFTDG-GGEYTLFPDGTPKPL 862
          :   * *             .   .: :*   *: **: .  .             :

MEDAKA KQDSHKSFNALRAYLL*FHTSGNMCFICKQLMH------------------MSRFLSGSE 1045
HUMAN  EEVTDEDIAAHGKFTIRFQPTTSTGIAEKSTLRDSTTEEKVPPITSTEGQVY-ATMEGSA 924
MOUSE  EKVSEEDLA-SGELTVTFHTSTSIGSAEKSASGEPTTGDRFLPTTSTEDQVINATAEGSA 921
       :: :.:.:       : *: : .     *.                          .**

MEDAKA LQVFL**RIFSP*RTF*VRGKGFA*LEEMQVLYLVIWQNCDQTLWFFHKVLMMHNHNPLI 1105
HUMAN  LGEVEDVDLSKP----VSTVPQFAHTSEVEGLAFVSYSSTQEPTT----YVDSSHTIPLS 976
MOUSE  LGE--DIEASKP----LFTGPPFVHTSDVEELAFVNYSSTQEPTT----YVDISHTSPLS 971
       *         .*          *.  .::: * :* :.. ::        :   :  **

MEDAKA AIPQCMWSVWWLLVYITVYYTH*KTQWCAQI*FFFLCIFIARNHVVPEFLFMCVTS*LTT 1165
HUMAN  VIPKTDWGVLVPSVPSEDEVLGEPSQDILVIDQTRL-----EATISPETMRT---TKITE 1028
MOUSE  IIPKTEWSVLETSVSLEDEILGKSDQDIL--EQTHL-----EATMSPGALRT---TGVSQ 1021
        **:  *.*    *           *         *     .  : *  :     : ::

MEDAKA RLAITGVCWQITATHRCTVDNTS*GDVIA-NSQSPLSVRVAGDCLTKAAHYSYFYCYSM* 1224
HUMAN  GTTQEEFPWKEQTAEKP-VPALSSTAWTPKEAVTPLDEQ-EGD----GSAYTVSEDELLT 1082
MOUSE  GETQEEP----QTPGSP-FPTFSSTAVMAKET---TAFE-EGE----GSTYTPSEGRLMT 1068
         :         :     .   *       ::      .  *:    .: *:      :

MEDAKA SAETLGLFAALPVFVWE--------NLVENLVALENSVLSGCVITKYIL*CQCAKNITEW 1276
HUMAN  GSERVPVLETTPVGKIDHSVSYPPGAVTEHKVKTDEVVTLTPRIGPKVSLSPGPE----- 1137
MOUSE  GSERVPGLETTPVGT-----SYPPGAITDQEVEMDTMVTLMSTIRPTVVSSTEPE----- 1118
       .:* :  : : **             :.:: *  :  *     *   :  .   :

MEDAKA TLSCAQKWWCYYELYPSGLD*LMTVQFTVYQVFIWNIMTWEIACLGCETQMSE----NNS 1332
HUMAN  ---------QKYETEGSSTTG-FTSSLSPFSTHITQLMEET-TTE--KTSLEDIDLGSGL 1184
MOUSE  ---------VIYEAEGSSPTE-FASTLRPFQTHVTQLMEET-TEEGKKASLDYTDLGSGL 1167
                  **   *.    ::  :  :...: ::*    :    ::.:.     ..
```

```
MEDAKA *KPPAIHSFCK*ICNISVCVVLAG*LSMTFLEMH*FTFKS*FMYGLHF-------KAEGK 1385
HUMAN  FEKPKATELIEF-STI--KVTVPSDITTAFSSVDRLHTTSAFKPSSAITKKPPLIDREPG 1241
MOUSE  FEP-RATELPKF-PTT------PSD-ISVFTAIDSLHRTPPLSPSSSFTEEQRVFEEESS 1218
        :     .: :   .        .    .*  :. :  .  :  .  :       . *

MEDAKA *KTKS--------------------------------FLIRLKNPYDGNHRAK------- 1406
HUMAN  EETTSDMVIIGESTSHVPPTTLEDIVAKETETDIDREYFTTSSPPATQPTRPPTVEDKEA 1301
MOUSE  EKTTGD-ILPGESVTQHPVTTLIDIVAMKTESDIDH---MTSKPPVTQPTRPSVVERKTT 1274
        :*..                                     . *     *

MEDAKA --------------RKL*--NFSYVLF*ND-------------DSISS-------RNDVQ 1430
HUMAN  FGPQALSTPQPPASTKFHPDINVYIIEVRENKTGRMSDLSVIGHPIDSESKEDEPCSEET 1361
MOUSE  SKTQELSTSTPAAGTKFHPDINVYIIEVRENKTGRLSDMIVSGHPIDSESKEEEPCSEET 1334
                      *:      *::  .:             . *.*        .:

MEDAKA DGWHRLLAIFSSVFA*ILTMSLTFSRLHIDEHFKKFYHNKLEEHL*FRVLYIKSHPKSKI 1490
HUMAN  DPVHDLMAEILPEFPDIIEIDL--------------YHSEENEEEEEECA-----NATDV 1402
MOUSE  DPLHDLFAEILPELPDSFEIDI--------------YHSEEDEDGEEDCV-----NATDV 1375
       *  * *:* :   :   : :.:              **.: :*.             :.:

MEDAKA EKKESKEYINLLVNI*KNIIKL*KQQQQXXXXXXXXXXXXXXXXXXXXXXXXXXXXXXXX 1550
HUMAN  TTTPSVQYINGKHLV----TTVPKDPEA-------------------------------- 1426
MOUSE  TTTPSVQYINGKQLV----TTVPKDPEA-------------------------------- 1399
        .. * :***    :     .: *: :

MEDAKA XXXXXXXXXXXXXXXXXXXXXXXXXXXXXXXXXXXXXXXXXXXXXXXXXXXXXXXXXXXX 1610
HUMAN  ------------------------------------------------------------
MOUSE  ------------------------------------------------------------


MEDAKA XXXXXXXXXXXXXXXXXXXXXXXXXXXXLKPQQEHNKFSH-QANPLMS*QN-----LKHP 1664
HUMAN  ------------------AEARRGQFESVAPSQNFSDSSESDTHPFVIAKTELSTAVQPN 1468
MOUSE  ------------------AEARRGQYESVAPSQNFPDSSATDTHQFILAETESSTTMQFK 1441
                                   : *.*:. . *  ::: ::  :.     ::

MEDAKA QLSYLQTPKD*VWKQVCHQANPQHVP--------QV------------------------ 1692
HUMAN  ESTETTESLEVTWKPETYPETSEHFSGGEPDVFPTVPFHEEFESGTAKKGAESVTERDTE 1528
MOUSE  KSKEGTELLEITWKPETYPETPDHVSSGEPDVFPTLSSHDG----KTTRWSESITESSPN 1497
       : .      : .**   :  . :*.          :

MEDAKA -------------LH--CSAQRSLHHCLSKKMILLL*T*QYCQLRHLVTKQKIILPLPQI 1737
HUMAN  VGHQAHEHTEPVSLFPEESSGEIAIDQESQKIAFARAT--EVTFGEEVEKSTSVTYTPTI 1586
MOUSE  LENPVHKQPKPVPLFPEESSGEGAIEQASQETILSRAT--EVALGKETDQSPTLST-SSI 1554
                    *.   *: .   .  *::  :   *     : . . :.  :     *

MEDAKA V**-----------ITLITP--KKRSSQRITSAVQ*NPFPVLTVELH--CPLK*LQCS-- 1780
HUMAN  VPSSASAYVSEEEAVTLIGNPWPDDLLSTKESWVEATPRQVVELSGSSSIPITEGSGEAE 1646
MOUSE  LSSSVSVNVLEEEPLTLTGISQTDESMSTIESWVEITPSQTVKFSESSSAPIIEGSGEVE 1614
       :             :**      .   .   * *: .*  .: ..     *:   . .

MEDAKA -APQKVLGMAQMISRKTHS*QQLCLQPME------QYHHLW*HLRTLKKVRLWEQPILLQ 1833
HUMAN  EDEDTMFTMVTDLSQRNTT---DTLITLDTSRIITE-SFFEVPATTIYPVS--EQ----- 1695
MOUSE  ENKNKIFNMVTDLPQRDPT---DTLSPLDMSKIMITNHHIYIPATTAPLDS--KL----- 1664
          :.:: *.  : ::  :     *  ::         .:     *       :

MEDAKA HCSAVQK*FHLKPQQEHNKF--SHQANLWMS*QNLKHPQLNY*QTPID*VWKQVCHQANP 1891
HUMAN  ----------PSAKVVPTKFVSETDTSEWISSTTVEEKKRKE-EEGT----------TGT 1734
MOUSE  ----------PSPDARPTQFGIQTATSEWVSDKSFEGRKREEDEEGA----------VNA 1704
                  . .   .:*  . :.  *:*  ..:  : :  :             ..
```

```
MEDAKA QHVPQVLHCSAQRSLHHCLSKKMILLLWT*QCCQLLHLLTKQKIILPLPQIVW*ITLIIP 1951
HUMAN  ASTFE-VYSSTQRSDQLIL-------PFELE---------SPNVATSSDSGTRKSFMSLT 1777
MOUSE  AHQGE-VRAATERSDHLLL-------TPELE---------SSNVDASSDLATWEGFILET 1747
           : : .:::** :  *           :         . ::       .    :

MEDAKA KKRSSQRITSAVQ*NPFPVLTVELHCPLK*LQCSAPQKVLGMAQMIHERLTHNSNCVXXX 2011
HUMAN  TPTQSERE----MTDSTPVFTET-----------------NTLENLGAQTTEHSSIHQ-PG 1816
MOUSE  TPTESEKE----MANSTPVFRET-----------------IGVANVEAQPFEHSSSSH-PR 1786
       .  .*::       :  **:                     : ::  :   *.*

MEDAKA XTDSGTSFSSKIASMLSTTESSGDGTDDFT--KDSLITAT-------VSSTQGTVSPSVV 2062
HUMAN  VQEGLTTLPRSPASVF-MEQGSGEAAADPETTTVSSFSLNVEYAIQAEKEVAGTLSPHVE 1875
MOUSE  VQEELTTLSGNPPSLF-TDLGSGDASTGMELITASLFTLDLESETKVKKELPSTPSPSVE 1845
         :  *::  .  *::    .**:.: .    . * ::          ..  .* ** *

MEDAKA ASLTPEKSETLGTASTAATLLSSTDKVLSETSARIQQIFTSSKPLDESTKPEASSIKLST 2122
HUMAN  TTFSTEP--------TGLVLSTVMDRVVAENITQTS------------------------ 1903
MOUSE  ISSSFEP--------TGLTPSTVLDIEIAGVMSQTS------------------------ 1873
        : : *         *. .  :  *  ::   :: .

MEDAKA DAQRLSVETGVPSSKPTARPTGSSLFSTEKPTSLFVKENDTVTVDLT-ILPVTPSGNQTE 2181
HUMAN  REIVISERLGEPNYGAEIRGFSTGFPLEEDFSGDF-REYSTVSHPIAKEETVMMEGSGDA 1962
MOUSE  QKTLISEISGKPTSQSGVRDLYTGFPMGEDFSGDF-SEYPTVSYPTMKEETVGMGGSDDE 1932
           :*   * *.     *   :.:   *. :. *  *  **:        *   *.

MEDAKA DYPS-STSDSVVDHLDHTQETFIPKDYVSSTVKSFSTTDSGKKSLPSVHMPSVKDTYTDM 2240
HUMAN  AFRDTQTSPSTVP-----------------------------TSVHISHISDSEGPSSTM 1993
MOUSE  RVRDTQTSSSIPT-----------------------------TSDNIYPVPDSKGPDSTV 1963
          . .** *                                .*     : . :.  : :

MEDAKA ESSTDLPDEESSSN-DESGSGLTEFTTESL------IEFIVTTDE----------AEINE 2283
HUMAN  VSTSAFPWEEFTSSAEGSGEQLVTVSSSVVPVLPSAVQKFSGTASSIIDEGLGEVGTVNE 2053
MOUSE  ASTTAFPWEEVMSSAEGSGEQLASVRSSVGPVLPLAVDIFSGTESPYFDEEFEEVAAVTE 2023
        *:: :* **  *. : **. *. . :.        :: :  * .          . :.*

MEDAKA TGSTLKIVST----------------ASSTYSLTLPSTQSS-HLAS-------------- 2312
HUMAN  IDRRSTILPTAEVEGTKAPVEKEEVKVSGTVSTNFPQTIEPAKLWSRQEVNPVRQEIESE 2113
MOUSE  ANERPTVLPTAASGNTVDLTENGYIEVNSTMSLDFPQTMEPSKLWSKPEVNLDKQEIGRE 2083
        .   .:: *                ...* *  :*.* .  :* *

MEDAKA ------------------------------------------------------KSLPLE 2318
HUMAN  TTSEEQIQEEKSFESPQNSPATEQTIFDSQTFTETELKTTDYSVLTTKKTYSDDKEMKEE 2173
MOUSE  TVTKEKAQGQKTFESLHSSFAPEQTILETQSLIETEFQTSDYSMLTTLKTYITNKEVEEE 2143
                                                             *.:  *

MEDAKA -QSSVDFSVDISSANSSESFTFKPSIILERKPFLSTAHTVDPQATFLYSTEKAIASSLDI 2377
HUMAN  DTSLVNMSTPDPDANGLESYTTLPEATEKSHFFLATALVTESIPAEHV----VTDSPIKK 2229
MOUSE  GMSIAHMSTPGPGIKDLESYTTHPEAPGKSHSFSATALVTESGAARSV----LMDSSTQE 2199
         * ..:*.   . :. **:*  *.   : : * :** ..:   :          *  .

MEDAKA HTSVNIFPTFHSTEKPSVPTAFKVSPFH---TVRTQSPPEILNLSRVQLLTEDKTSGDPA 2434
HUMAN  EESTKHFPKGMRPTIQESDTELLFSGLGSGEEVLPTLPTESVNFTEVEQINNTLYPHT-- 2287
MOUSE  EESIKLFQKGVKLTNKESNADLSFSGLGSGEA-LPPLPTTSVNLTDMKQIISTLYAET-- 2256
       . * : * .       .  : : .* :          *   :*:: :: : .

MEDAKA NESFISKLLVLGNFTSSTPTGKTESLPALDNLSFS---LEGETAEYTKDS-LISQATDSS 2490
HUMAN  -----SQVEST-------SSDKIEDFNRMENVAKEVGPLVSQTDIFEGSGSVTSTTLIEI 2335
MOUSE  -----SHMESLGT---SILGDKMEDHERMEDVSNEVRMLISKTGSISQDS---TEALDTT 2305
            *::            .* *.   ::::: .   * .:*     ..   : :
```

```
MEDAKA TGNDGANLPRNTLHPSHTASLLTPESLKSDQTVPDFTSETVSSLKDKVSSIPTAFPTIIY 2550
HUMAN  LSDTGAEGPTVA--------------------------------------PLPFST--- 2353
MOUSE  LSHTGTEEPTTS--------------------------------------TLPFVKLM- 2325
        .. *:: *  :                                          * .

MEDAKA RSIADQQVGIITPSTKQVETIKAGQTPTMVLHEPKASISVYKIFTEEAKENNELISGGTE 2610
HUMAN  ---------------------DIGHPQ-------NQTV----RWAEEIQ----------- 2370
MOUSE  ---------------------DLERSP-------KQTL----RWEEETQ----------- 2342
                            .  :         : ::     : ** :

MEDAKA SLTPESITSEFITKDNAI--I---DTISTAQVPFFYPTVLTSGGIKADTKAQKLKIMEET 2665
HUMAN  TSRPQTITEQDSNKNSSTAEINETTTSSTDFLARAYGFEMAKEFVTSAPKPSDLYYEPSG 2430
MOUSE  THRPQTMSGLISNENSSASEAEEAATSPTAFLPQTYSVEMTKHFAPSESQPSDLFNVNSG 2402
       :  *::::    .::.:        *  *  :   *   ::.    :  : ..*    .

MEDAKA EGSGTARSAILTPTPSILSAAPKSESVTLTSHGIISTM---------------------- 2703
HUMAN  EGSGEVDIVDSFHTSATTQATRQESSTTFVSDGSLEKHPEVPSAKAVTADGFPTVSVMLP 2490
MOUSE  EGSGEVDTLDLVYTSGTTQASSQG-DSMLASHGFLEKHPEVSKTEAGATDVSPTASAMFL 2461
       **** .       * .  .*: :  .  :.*.* :..

MEDAKA ----------------------------------RTERD----SA-----KT--------- 2712
HUMAN  LHSEQNKSSPDPTSTLSNTVSYERSTDGSFQ--DRFREFEDSTLKPNRKKPTENIIIDLD 2548
MOUSE  HHS-EYKSSLYPTSTLPSTEPYKSPSEGIEDGLQDNIQFEGSTLKPSRRKTTESIIIDLD 2520
                                            :    :      *

MEDAKA SENYDDLSVKMITSSVYSMFS-------------TQKPTLDTKDIVPGDLDQSIFTLSTA 2759
HUMAN  KED-KDLILTITESTILEILPELTSDKNTIIDIDHTKPVYEDILGMQTDIDTEVPSEPHD 2607
MOUSE  KEDSKDLGLTITESAIVEILPELTSDKNIIIDIDHTKPVYEYIPGIQTDLDPEIKLESHG 2580
       .*: .** :.:  *:: .::                **. :    :  *:* .:

MEDAKA DS-NISENTTLLSETLETETPVSIPEETFKTTEKD----ETQTLHSSGVSEQ-KKEF--L 2811
HUMAN  SNDESNDDSTQVQEIYEAAVNLSLTEETFEGSADV-LASYTQATHDESMTYEDRSQLDHM 2666
MOUSE  SS----EESLQVQEKYEGAVTLSPTEESFEGSGDALLAGYTQAIYNESVTPNDGKQAEDI 2636
       ..    :::  :.*  *  . :*  **:*: : .      **: :...:: :  .:   :

MEDAKA PIGRITSAPTHEEITSSVEISPNASTVSFPQSTPKSKVTVQFVTTFALQPDTIQTVETFQ 2871
HUMAN  GFHFTTGIPAPSTETELDVLLPTATSLPIPRKSATV------------------------ 2702
MOUSE  SFSFATGIPVSSTETELHTFFPTASTLHIPSKLTTA------------------------ 2672
        :   *. *. .  *.   : *.*::: :* .  .

MEDAKA HARSEPHQFRNDSSLESLKQVRSEIQSTQTPQANHNSQDASLTTILPISTSHQGSQITKF 2931
HUMAN  ---------------------IPEIE-------GIKAEAKALDDMFESSTLSDGQAIADQ 2734
MOUSE  ---------------------SPEID-------KPNIEAISLDDIFESSTLSDGQAIADQ 2704
                              **:         : :  :*  ::  **  :*. *:.

MEDAKA TAIHPGDHSVTEAGSVLEDGKTLKLKTKPSSDTKYI--------DNIDYTAPD------- 2976
HUMAN  SEIIPTLGQFERTQEEYEDKK----HAGPSFQPEFSSGAEEALVDHTPYLSIATTHLMDQ 2790
MOUSE  SEVISTLGHLEKTQEEYEEKK----YGGPSFQPEFFSGVGEVLTDPPAYVSIGSTYLIAQ 2760
       : :      . .: .  *: *       ** : ::         *   * :

MEDAKA --YDLVDPIRLESVPKYKNNSKEMEDSLAKPQTPISTSPISFYESGSESTSSSEESMPLT 3034
HUMAN  SVTEVPDVMEGSNPPYYTDT-TLAVSTFAKLSSQTPSSPLTIYSGSEASGH---TEIPQP 2846
MOUSE  TLTELPNVVRPSDSTHYTEA-TPEVSSLAELSPQIPSSPFPVYVDNGVSKF---PEVPHT 2816
          :: : :. ..   *.:  .   .::*: .    :**: .* ..  *      .:*

MEDAKA STAKV-----------------------------DSYVRGKIPSDLLSPTTTMSSVLDPG 3065
HUMAN  SALPGIDVGSSVMSPQDSFKEIHVNIEATFKPSSEEYLHITEPPS-LSPDTKLEPSEDDG 2905
MOUSE  SAQPVSTVTSSQKSIESPFKEVHANIEETIKPLGG-NVHRTEPPS-MSRDPALDVSEDES 2874
       *:                                   :: . * . :*    :.   * .
```

```
MEDAKA LQSVNSLTMSVKPYKE----NTRRL-----EEQLRLTDEILAVFKEYSTAASRTET-FNS 3115
HUMAN  KPELL-EEMEASPTELIAVEGTEILQDFQNKTDGQVSGEAIKMFPTIKTPEAGTVITTAD 2964
MOUSE  KHKLL-EELETSPTK------PETSQDFPNKAKDHIPGETVGMLAGIRTTESEPVI-TAD 2926
         .:    :...* :       .       : . :: .* : ::    *  :       .

MEDAKA DNTLGNVQNL-------STVPMENSEITSRIPAKSASSTTQSMQE----SATSVATTA-L 3163
HUMAN  EIELEGATQWPHSTSASATYGVEAGVVPWLSPQTSERPTLSSSPEINPETQAALIRGQDS 3024
MOUSE  DMELGGATQQPHSA--SAAFRVETGMVPQPIQQEPERPTFPSL-EINHETHTSLF--GES 2981
       :  * .. :         ::  :* . :           *  *  *    : :::

MEDAKA PIPVEDGKQPGDLLSSVTEG--GQSLEKK-EKSTPPPVAL----------D-LGHTVVGE 3209
HUMAN  TIAASEQQVAARILDSNDQATVN-PVEFNTEVATPPFSLLETSNETDFLIGINEESVEGT 3083
MOUSE  ILATSEKQVSQKILDNSNQATVSSTLDLHTAHALSPFSILDNSNETAFLIGISEESVEGT 3041
        : ..: :    :*..  :.  .  :: :   :  *   *          .   .:* *
```

G3 domain

```
MEDAKA TMEIPGLYSCAKNICLNGGSCYKSGSVLSCSCAPGYTGPLCENDIDECHSNPCRNGGTCV 3269
HUMAN  AIYLPGPDRCKMNPCLNGGTCYPTETSYVCTCVPGYSGDQCELDFDECHSNPCRNGATCV 3143
MOUSE  AVYLPGPDLCKTNPCLNGGTCYPTETSYVCTCAPGYSGDQCELDFDECHSNPCRNGATCV 3101
       :: :**   *  * *****:** : :    *:*.***:*  ** *:***********.***

MEDAKA DGLASFSCVCLPSYTGLYCESDTETCEYGWHKFQGHCYKHFPQRKNWDSAERECRMQGAH 3329
HUMAN  DGFNTFRCLCLPSYVGALCEQDTETCDYGWHKFQGQCYKYFAHRRTWDAAERECRLQGAH 3203
MOUSE  DGFNTFRCLCLPSYVGALCEQDTETCDYGWHKFQGQCYKYFAHRRTWDAAERECRLQGAH 3161
       **: :* *:*****.*  **.*****:********:***:* :*:.**:******:****

MEDAKA LTSILSHEEQLFVNRLGQDYQWIGLNDKVFQNDFRWTDGSVVQYEHWRPNQPDSFFSSGE 3389
HUMAN  LTSILSHEEQMFVNRVGHDYQWIGLNDKMFEHDFRWTDGSTLQYENWRPNQPDSFFSAGE 3263
MOUSE  LTSILSHEEQMFVNRVGHDYQWIGLNDKMFEHDFRWTDGSALQYENWRPNQPDSFFSAGE 3221
       **********:****:*:**********:*::********.:***:***********:**

MEDAKA DCVVLIWHEDGQWNDVPCNYHLTYTCKKGTVACSQPPVVENARTYGKKRERYEINSLVRY 3449
HUMAN  DCVVIIWHENGQWNDVPCNYHLTYTCKKGTVACGQPPVVENAKTFGKMKPRYEINSLIRY 3323
MOUSE  DCVVIIWHENGQWNDVPCNYHLTYTCKKGTVACGQPPVVENAKTFGKMKPRYEINSLIRY 3281
       ****:****:***********************.********:*:** : *******:**

MEDAKA RCRTGFIQRHVPTIRCRGDGRWDVPKITCLNPSSYQRSFIRRHQHQHRSL-----YSVNN 3504
HUMAN  HCKDGFIQRHLPTIRCLGNGRWAIPKITCMNPSAYQRTYSMKYFKNSSSAKDNSINTSKH 3383
MOUSE  HCKDGFIQRHLPTIRCLGNGRWAMPKITCMNPSAYQRTYSKKYLKNSSSAKDNSINTSKH 3341
       :*: ******:***** *:*** :*****:***:***::  :: ::  *       : ::

MEDAKA FKKWQEDSFHSGYRRYRGRRDRNEHKRKKVVSSK* 3539
HUMAN  DHRWSR--------------RWQESRR-------- 3396
MOUSE  EHRWSR--------------RRQETRR-------- 3354
        ::*..                :* :*
```

Fig. S3

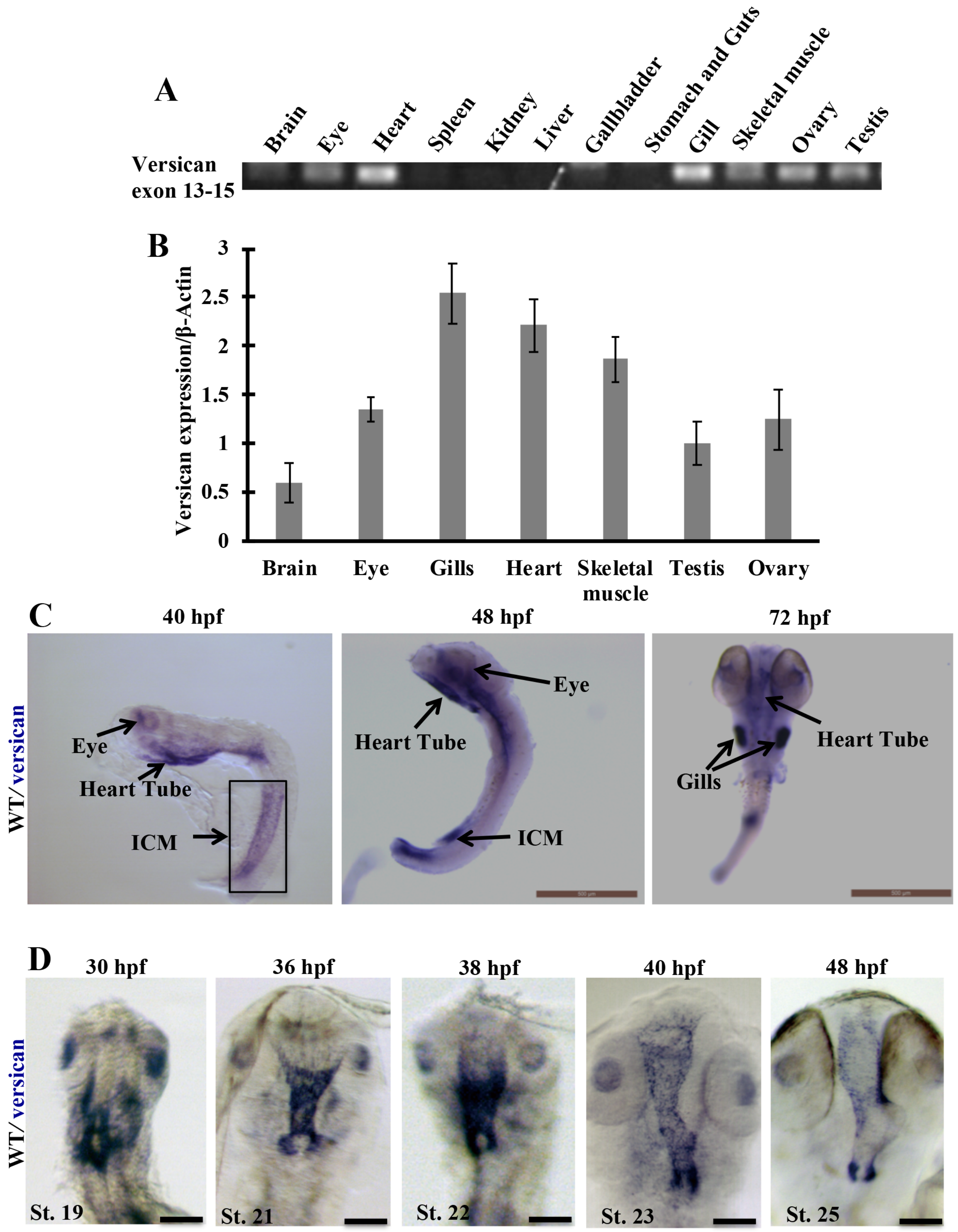
A
Brain
Eye
Heart
Spleen
Kidney
Liver
Gallbladder
Stomach and Guts
Gill
Skeletal muscle
Ovary
Testis
Versican
exon 13-15
B
Versican expression/β-Actin
3
2.5
2
1.5
1
0.5
0
Brain
Eye
Gills
Heart
Skeletal muscle
Testis
Ovary
C
40 hpf
48 hpf
72 hpf
WT/ versican
Eye
Heart Tube
ICM
Eye
Heart Tube
ICM
Heart Tube
Gills
D
30 hpf
36 hpf
38 hpf
40 hpf
48 hpf
WT/ versican
St. 19
St. 21
St. 22
St. 23
St. 25

Fig. S4

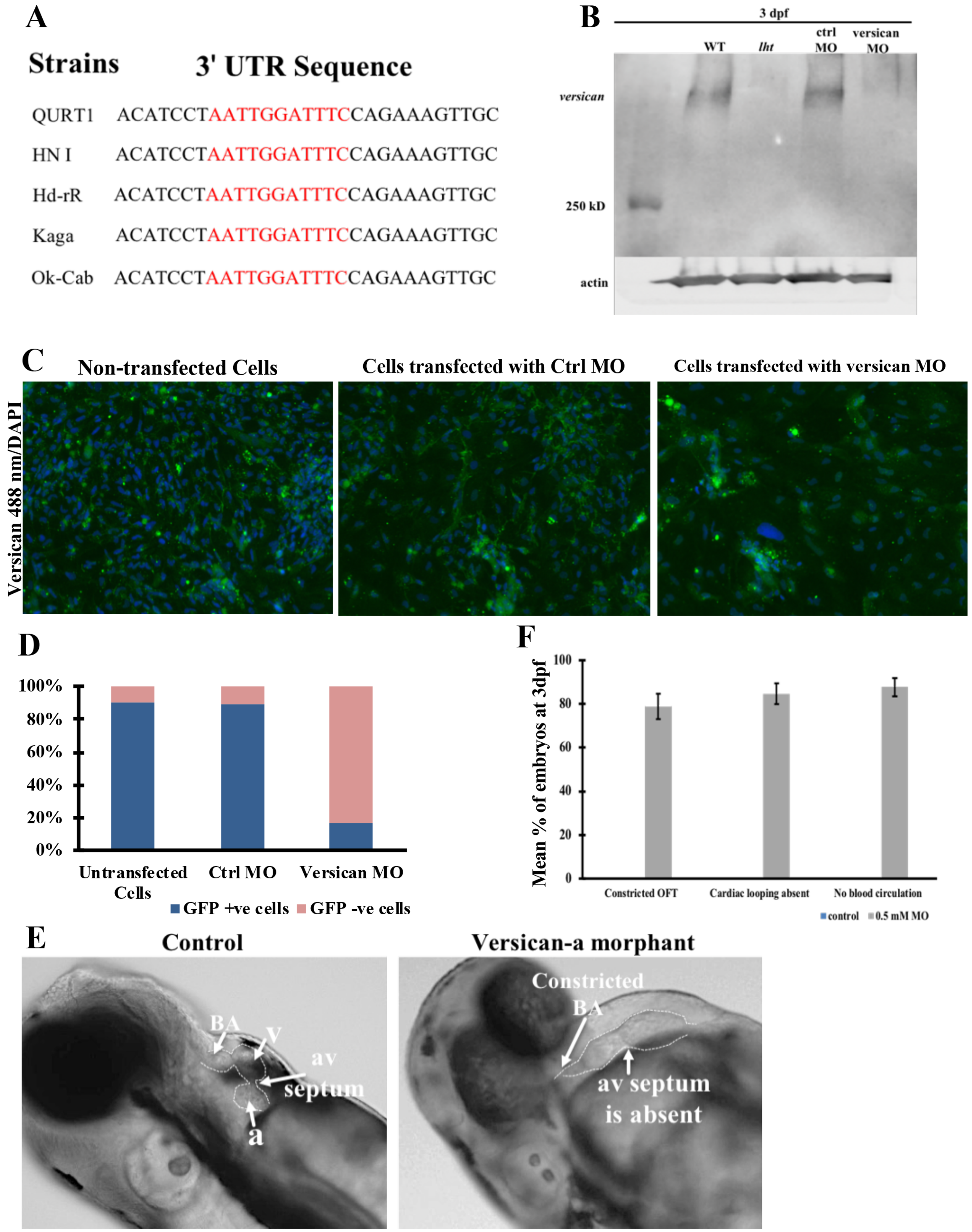
A
Strains
3' UTR Sequence
QURT1 ACATCCTAATTGGATTTCCAGAAAGTTGC
HN I ACATCCTAATTGGATTTCCAGAAAGTTGC
Hd-rR ACATCCTAATTGGATTTCCAGAAAGTTGC
Kaga ACATCCTAATTGGATTTCCAGAAAGTTGC
Ok-Cab ACATCCTAATTGGATTTCCAGAAAGTTGC
B
3 dpf
WT
lht
ctrl MO
versican MO
versican
250 kD
actin
C
Non-transfected Cells
Cells transfected with Ctrl MO
Cells transfected with versican MO
Versican 488 nm/DAPI
D
100%
80%
60%
40%
20%
0%
Untransfected Cells
Ctrl MO
Versican MO
GFP +ve cells
GFP -ve cells
F
Mean % of embryos at 3dpf
100
80
60
40
20
0
Constricted OFT
Cardiac looping absent
No blood circulation
control
0.5 mM MO
E
Control
Versican-a morphant
BA
V
av septum
a
Constricted BA
av septum is absent

Fig. S5

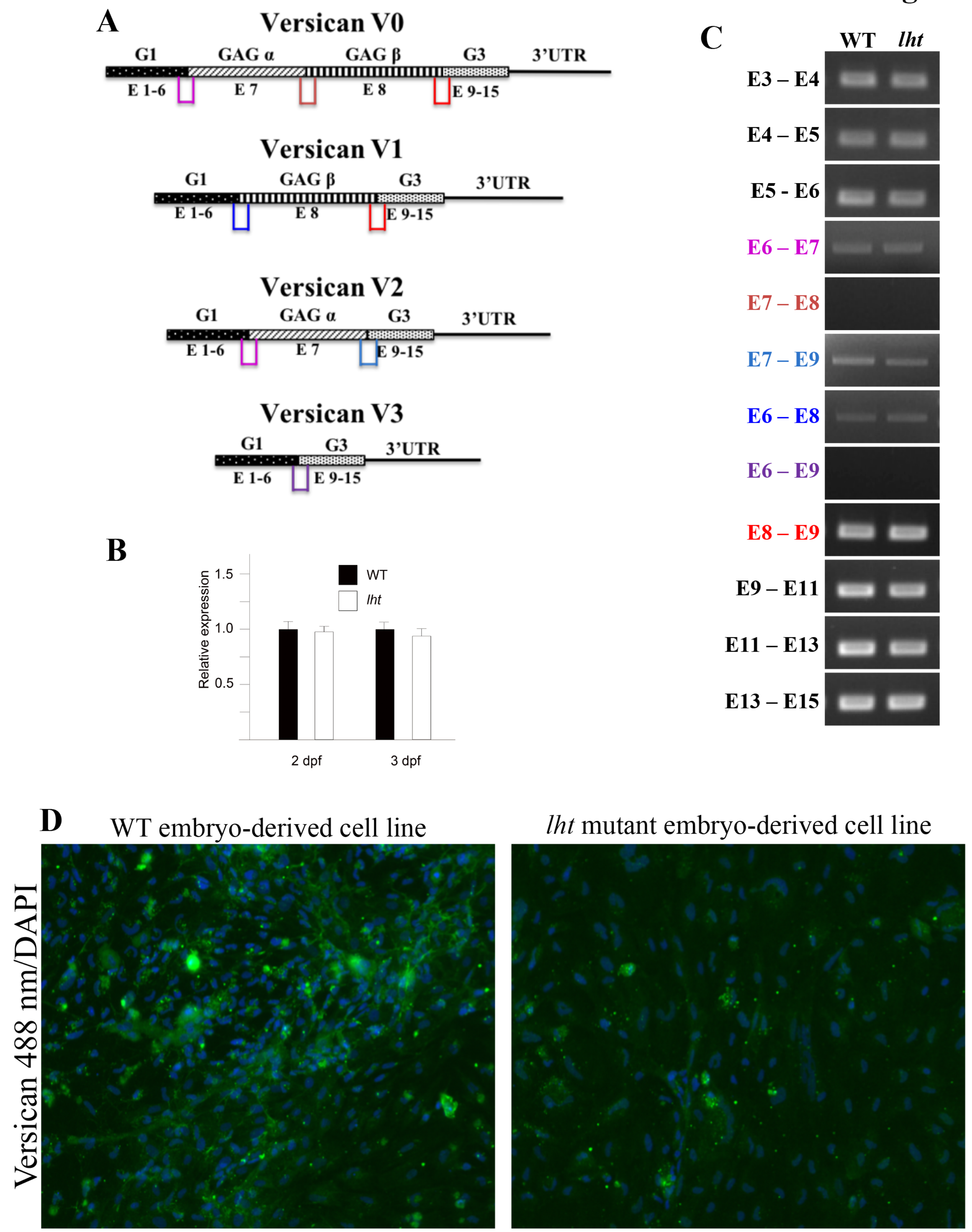
A
Versican V0
G1
GAG α
GAG β
G3
3'UTR
E 1-6
E 7
E 8
E 9-15
Versican V1
G1
GAG β
G3
3'UTR
E 1-6
E 8
E 9-15
Versican V2
G1
GAG α
G3
3'UTR
E 1-6
E 7
E 9-15
Versican V3
G1
G3
3'UTR
E 1-6
E 9-15
B
Relative expression
1.5
1.0
0.5
WT
lht
2 dpf
3 dpf
C
WT
lht
E3 – E4
E4 – E5
E5 - E6
E6 – E7
E7 – E8
E7 – E9
E6 – E8
E6 – E9
E8 – E9
E9 – E11
E11 – E13
E13 – E15
D
WT embryo-derived cell line
lht mutant embryo-derived cell line
Versican 488 nm/DAPI

Fig. S6

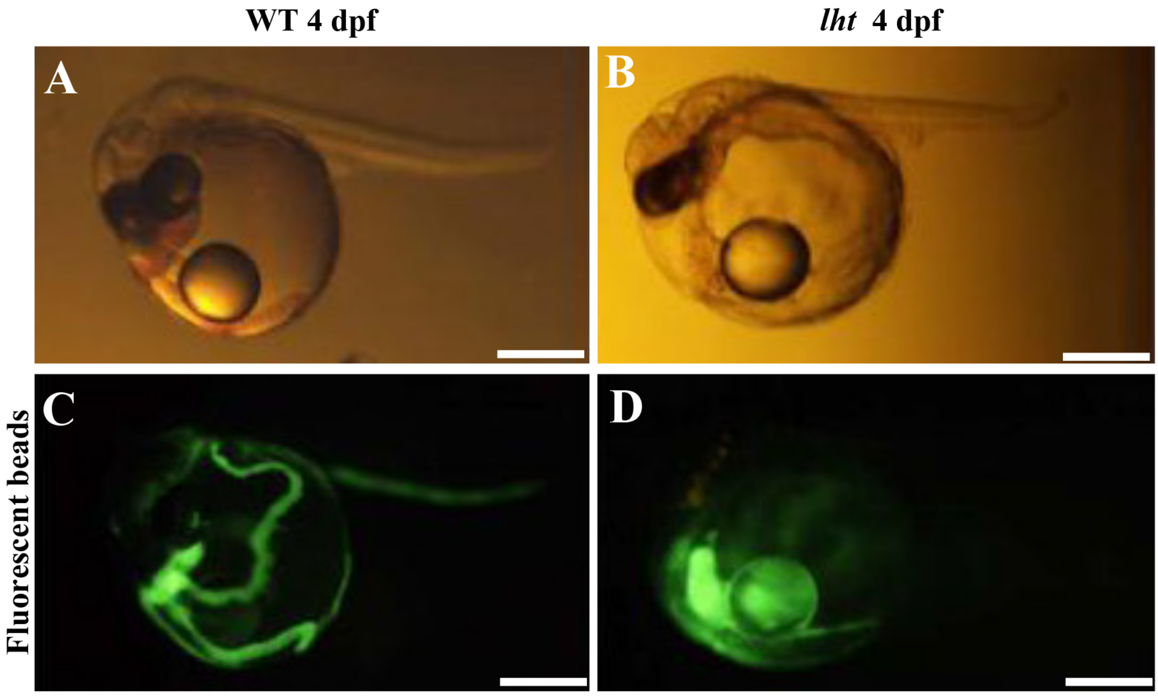

Fig. S7

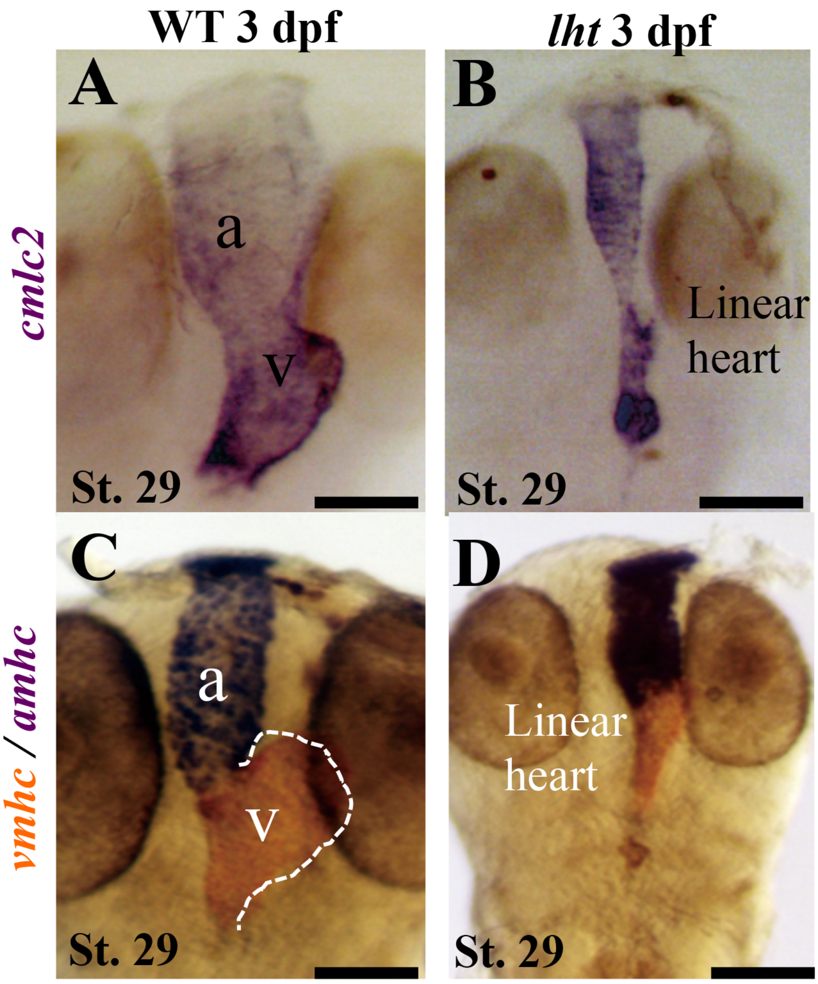

WT 3 dpf
lht 3 dpf
A
a
V
St. 29
B
Linear
heart
St. 29
C
a
V
St. 29
D
Linear
heart
St. 29
cmlc2
vmhc / *amhc*

Fig. S8

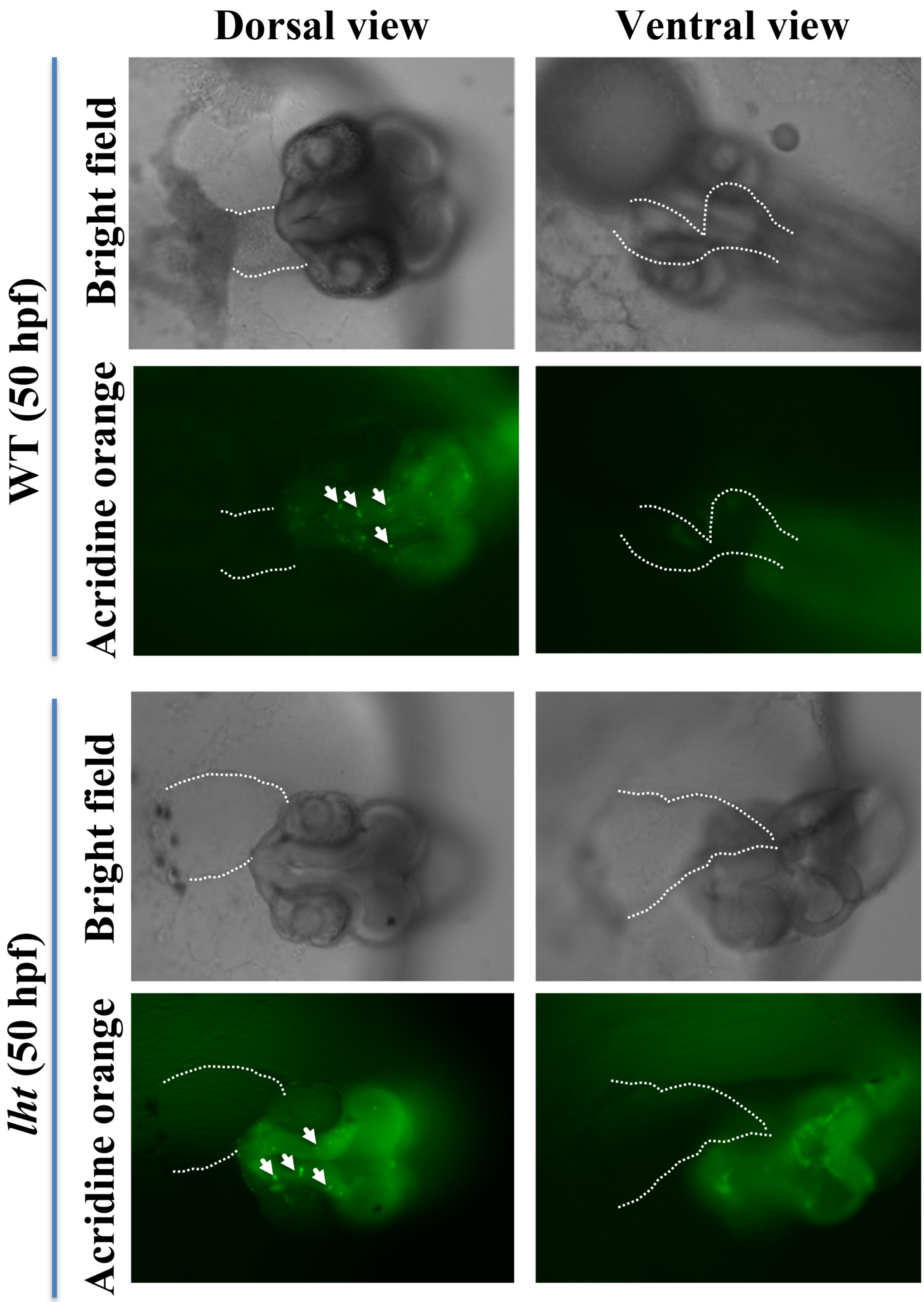

Dorsal view
Ventral view
WT (50 hpf)
Bright field
Acridine orange
lht (50 hpf)
Bright field
Acridine orange

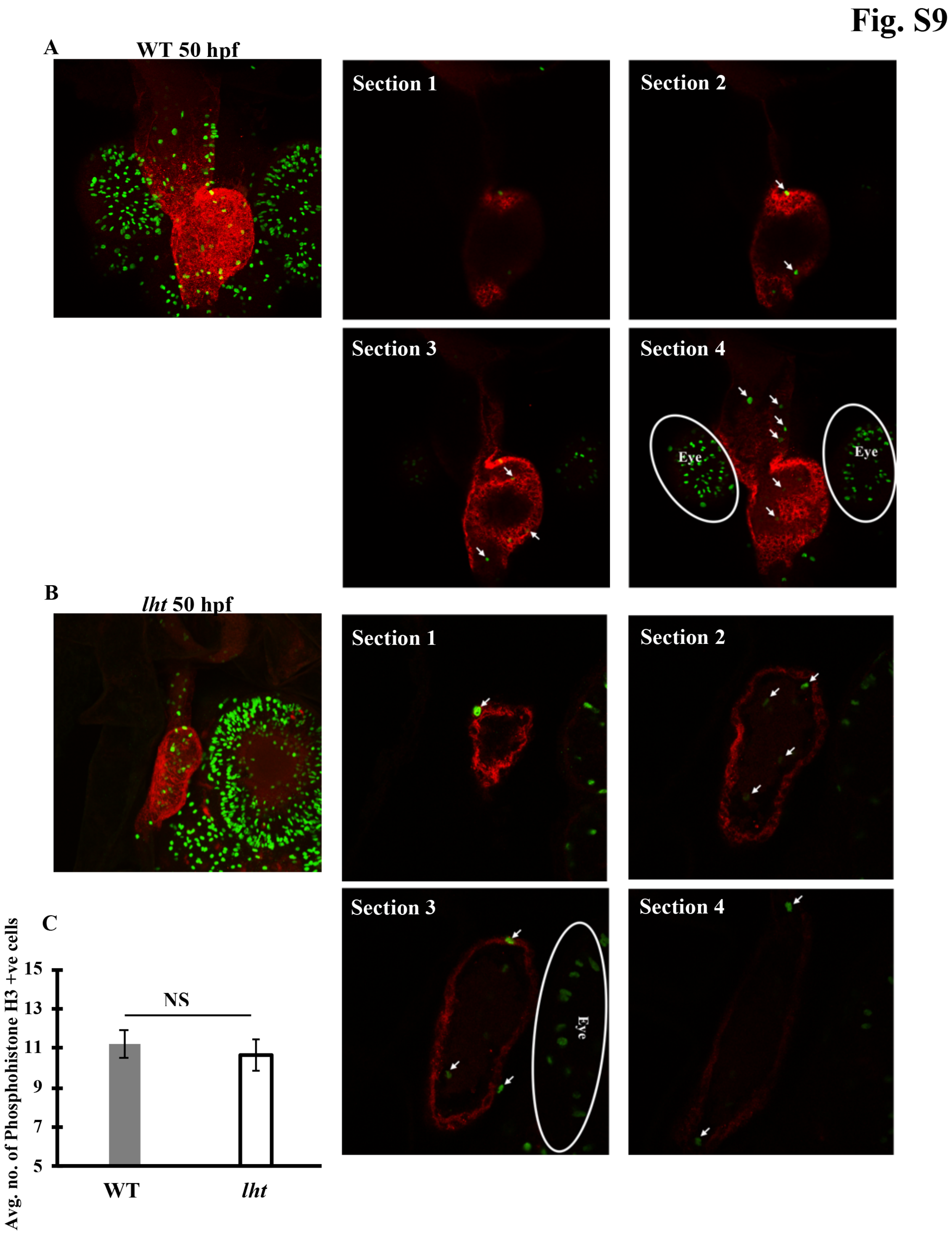

Fig. S9
A
WT 50 hpf
Section 1
Section 2
Section 3
Section 4
Eye
Eye
B
lht 50 hpf
Section 1
Section 2
Section 3
Eye
Section 4
C
Avg. no. of Phosphohistone H3 +ve cells
15
13
11
9
7
5
NS
WT
lht

Fig S10

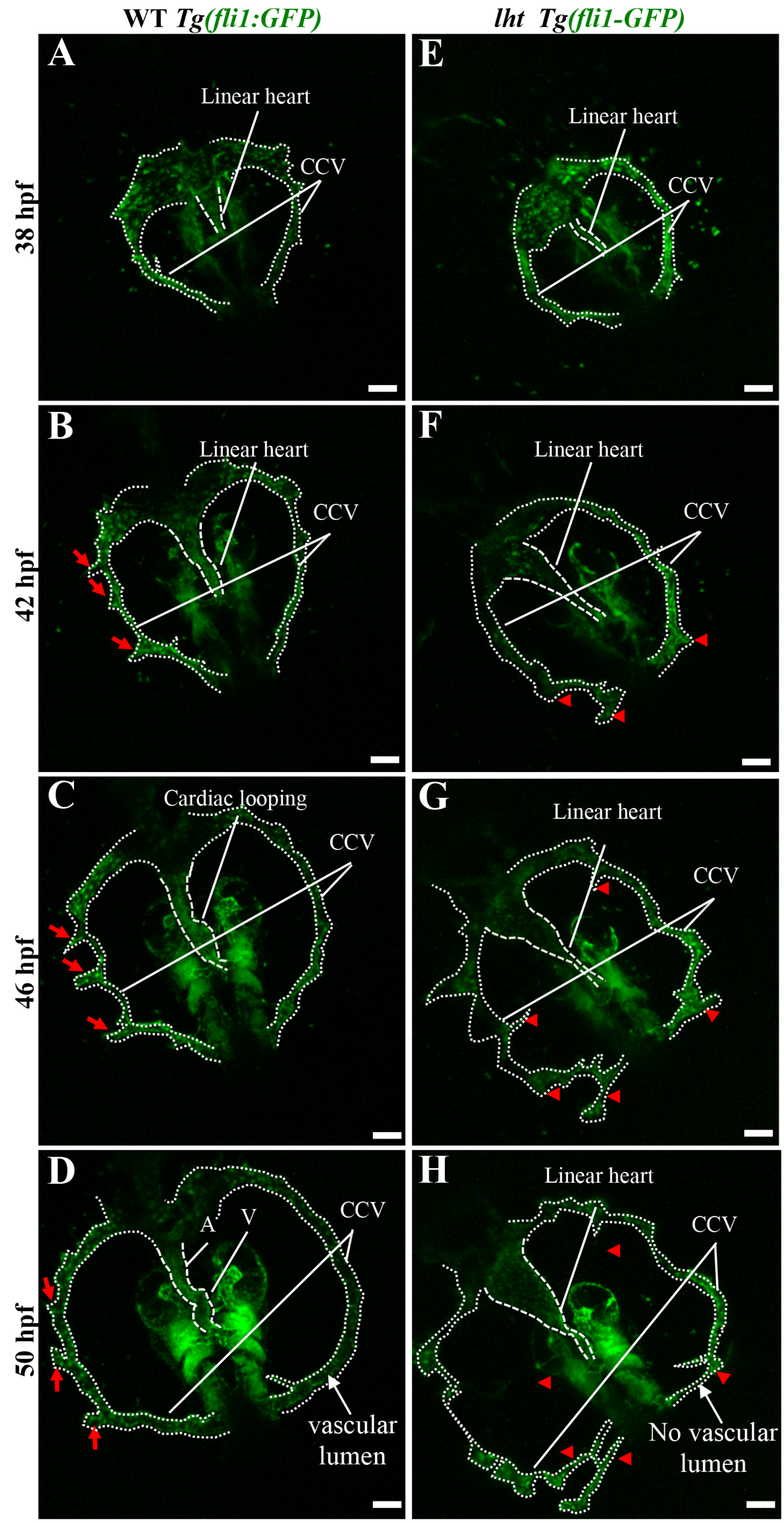
WT Tg(fli1:GFP)
lht Tg(fli1-GFP)
38 hpf
42 hpf
46 hpf
50 hpf
A
Linear heart
CCV
B
Linear heart
CCV
C
Cardiac looping
CCV
D
A
V
CCV
vascular lumen
E
Linear heart
CCV
F
Linear heart
CCV
G
Linear heart
CCV
H
Linear heart
CCV
No vascular lumen

Fig. S11

WT 2.3 dpf (after yolk removal)

etgase

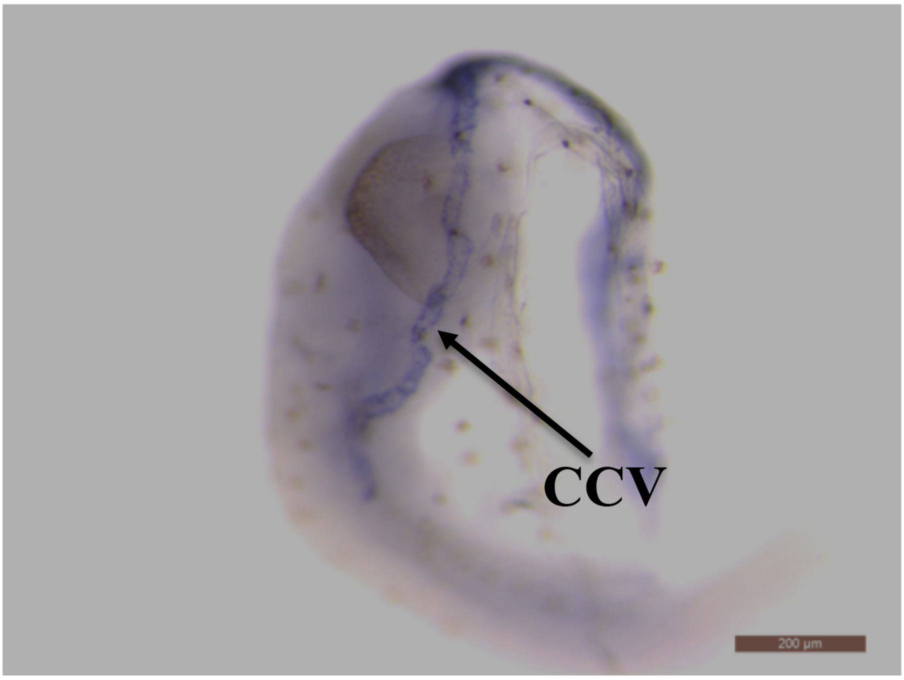

Fig. S12

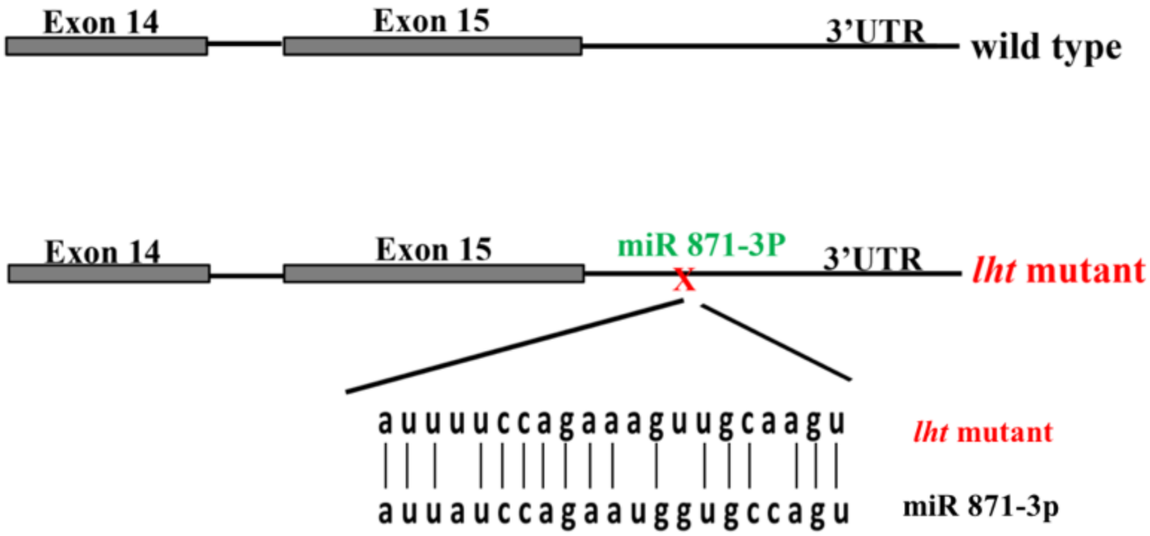

Exon 14
Exon 15
3'UTR
wild type
Exon 14
Exon 15
miR 871-3P
X
3'UTR
lht mutant
a u u u u c c a g a a a g u u g c a a g u
lht mutant
a u u a u c c a g a a u g g u g c c a g u
miR 871-3p

Fig. S13

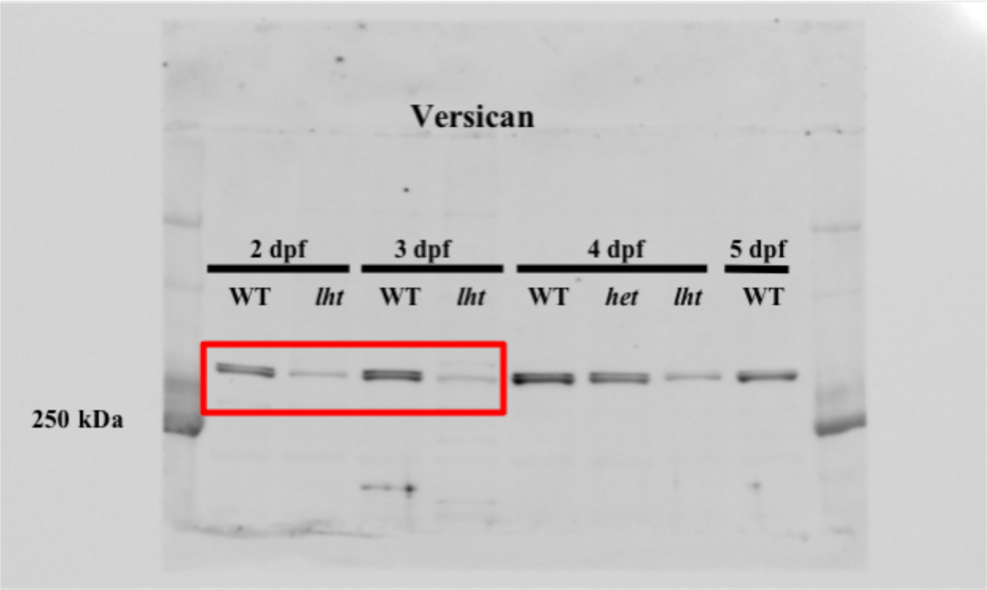

Versican
2 dpf
3 dpf
4 dpf
5 dpf
WT
lht
WT
lht
WT
het
lht
WT
250 kDa

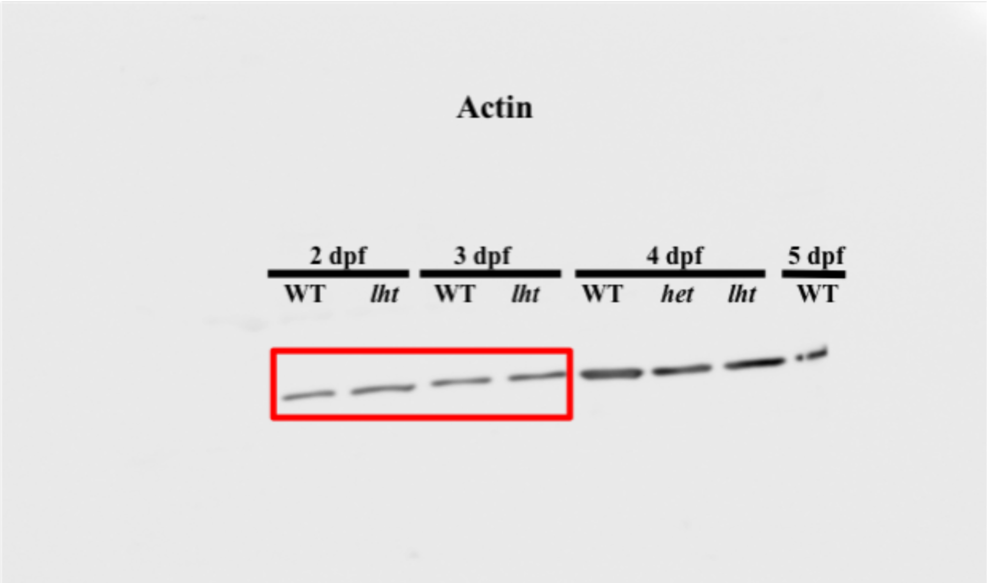

Actin
2 dpf
3 dpf
4 dpf
5 dpf
WT
lht
WT
lht
WT
het
lht
WT

Fig. S14

Versican

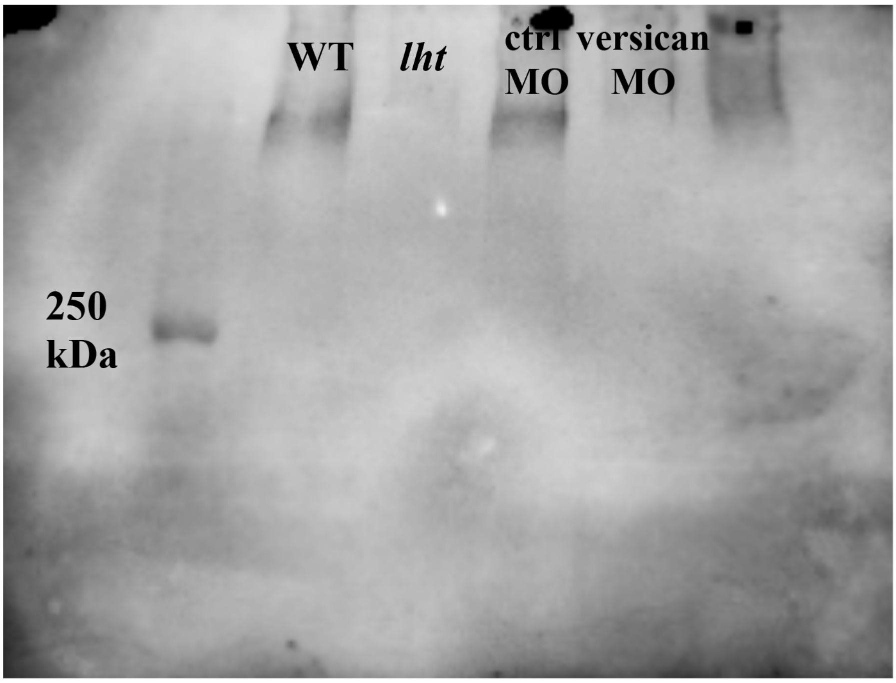

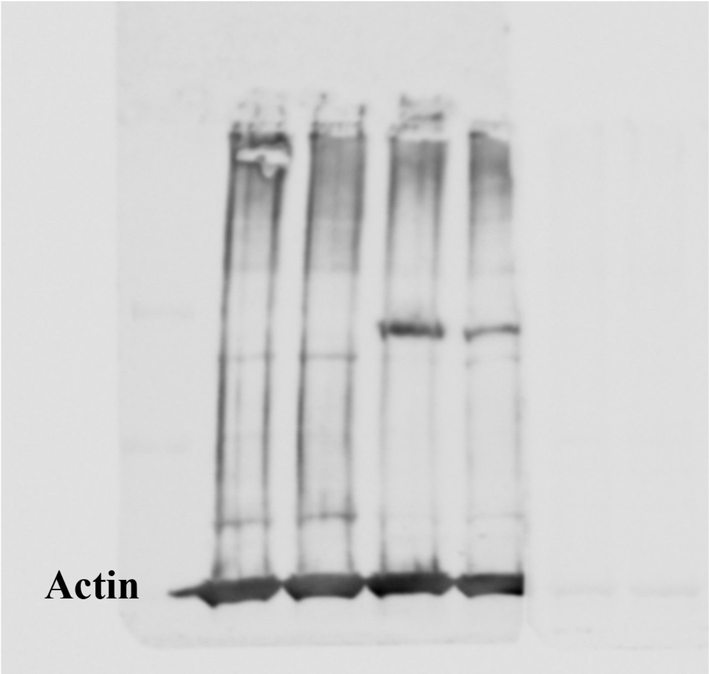

TABLE 1: Primers used for mapping

Contig. Name	Forward Primer (5'--------3')	Reverse Primer (5'--------3')	Restriction Enzyme
Olb0807j	TCGACACCTTCTGTTTGTGAGAGCG	GGCCTTAGCAGTTGTTTTCTGGCA	Hinfl
MFOISSA038C07	CCTGTCGTTGCTGAGTCCTTC	AGGTGTAATGCTGTTCGACCTC	RSaI
Ol11523	ATTTAGTCCCCCAGGAGTGG	CGTTCTCGTTTCTCCTGCAA	RSaI
Ol12320	GCAGGTGGAAGTCGACAAA	CCAATGGACCATCACTTCC	RSaI
Ol12338i5	TCCTTCAAAGGGAGTGATGG	ACCCAGAGGTGAAGACATGC	Hinfl
Ol12419	AGTCAAGGGCAAGGATGATG	AGAGTTGGCTGATGGAGCAT	Hae3
Ol1262li4	TCTGCCATCTCTACTTCTTATGG	TGAGGACAGGTCTGCACTGTT	Hinfl
Ol102119	CCAGCCAGAAGAACAAATGC	GCGAAAGCCCTGTTAAAACT	RSaI
TiT8j07	TGAGCATTGGGTTTACTGTG	ACTTGTTGCCGCTTGAGATG	RSaI

TABLE 2: Primers used to verify splicing of versican gene

Primer Name	Forward Primer (5'--------3')	Primer Name	Reverse Primer (5'--------3')
Vcan ex 3	CAGTGCCCAGTCATCCTGAA	Vcan ex 4	ATCTTGACAGGCCCGAACAG
Vcan ex 4	CTGTTCGGGCCTGTCAAGA	Vcan ex 5	TGCCAGGGTTAGTCATGAGG
Vcan ex 5	CCTCATGACTAACCCTGGCA	Vcan ex 6	CCATAGTCGCATCTGTCTAGC
Vcan ex 6	CCAGAATTCAGTGTGGGGGA	Vcan ex 7	TGGATGGAGCCATGCTAGTG
Vcan ex 7	CTACCACAATAAGTTAGAGGAACAT	Vcan ex 8	AGACTTAGCAGGGATCCTCG
Vcan ex 7	CTACCACAATAAGTTAGAGGAACAT	VCan ex 9	AAGAGGACCAGTGTAACCGG
Vcan ex 6	ATGCCCTTGACAAGCACTGC	Vcan ex 8	AGACTTAGCAGGGATCCTCG
Vcan ex 6	ATGCCCTTGACAAGCACTGC	Vcan ex 9	AAGAGGACCAGTGTAACCGG
Vcan ex 8	GATGGCAAACAACCTGGCGA	Vcan ex 9	AAGAGGACCAGTGTAACCGG
Vcan ex 9	CCGGTTACACTGGTCCTCTT	Vcan ex 11	CATTCTCGCTCTGCTGAGTC
Vcan ex 11	GACTCAGCAGAGCGAGAATG	Vcan ex 13	CGTCATTCCACTGTCCATCC
Vcan ex 13	GGATGGACAGTGGAATGACG	Vcan ex 15	GCTCCTCTGGTAGCTTGAAG

TABLE 3: Primers for real-time PCR

Primer Name	Forward Primer (5'--------3')	Reverse Primer (5'--------3')
BNP	CTTCATCCCTTTTTGGGGGC	TTCTCGCTCCTCCTCTGTG
Versican	GGATGGACAGTGGAATGACG	GCTCCTCTGGTAGCTTGAAG

TABLE 4: Primers for in-situ Probe design

Primer Name	Forward Primer (5'--------3')	Reverse Primer (5'--------3')
Cmlc2	AATGTCTTTTCCATGTTTGAGC	CTCCTCTTTCTCATCCCCATG
Amhc	TGCACTGATGGCTGAATTTG	ACTTGATCTACA CCTTGGCC
Vmhc	GCTGAGATGTCCGTGTATGGTGC	GCTCCTCACGAGGCCTCTGCTTG
Bnp	GATCCATCCATCCATCATCC	TGATACTTTAAAGACACAATGTCCAA
Versican	CACGACAATTCTCCCCATCT	TGCTCTTCCAGTCTCCTGGT
e-tgase	CCCACCCCTGACAGAGTTTC	GTCACAACACGAGACGGGAT
TMEM 205	GCTCTACGTCTGAACTTGCG	ATCTCATGCCAGCTTGTTCAAA
Egfl7	GCACCTACAAGACCACCTAC	GGGACGAGGAGAATCACGAA
